# Humanin selectively prevents the activation of pro-apoptotic protein BID by sequestering it into fibers

**DOI:** 10.1074/jbc.RA120.013023

**Published:** 2025-01-13

**Authors:** Daniel L. Morris, Sabrina Johnson, Christopher K.E. Bleck, Duck-Yeon Lee, Nico Tjandra

**Affiliations:** 1 Laboratory of Molecular Biophysics, Biochemistry and Biophysics Center, NHLBI, National Institutes of Health, Bethesda, Maryland, USA; 2 Electron Microscopy Core Facility, NHLBI, National Institutes of Health, Bethesda, Maryland, USA; 3 Biochemistry Core Facility, NHLBI, National Institutes of Health, Bethesda, Maryland, USA

**Keywords:** apoptosis, BID, humanin, fibers, conformational change, β-sheet, amyloid, electron microscopy (EM), B-cell lymphoma 2 (Bcl-2) family

## Abstract

Members of the B-cell lymphoma (BCL-2) protein family regulate mitochondrial outer membrane permeabilization (MOMP), a phenomenon in which mitochondria become porous and release death-propagating complexes during the early stages of apoptosis. Pro-apoptotic BCL-2 proteins oligomerize at the mitochondrial outer membrane during MOMP, inducing pore formation. Of current interest are endogenous factors that can inhibit pro-apoptotic BCL-2 mitochondrial outer membrane translocation and oligomerization. A mitochondrial-derived peptide, Humanin (HN), was reported being expressed from an alternate ORF in the mitochondrial genome and inhibiting apoptosis through interactions with the pro-apoptotic BCL-2 proteins. Specifically, it is known to complex with BAX and BID. We recently reported the fibrillation of HN and BAX into β-sheets. Here, we detail the fibrillation between HN and BID. These fibers were characterized using several spectroscopic techniques, protease fragmentation with mass analysis, and EM. Enhanced fibrillation rates were detected with rising temperatures or pH values and the presence of a detergent. BID fibers are similar to those produced using BAX; however, the structures differ in final conformations of the BCL-2 proteins. BID fibers display both types of secondary structure in the fiber, whereas BAX was converted entirely to β-sheets. The data show that two distinct segments of BID are incorporated into the fiber structure, whereas other portions of BID remain solvent-exposed and retain helical structure. Similar analyses show that anti-apoptotic BCL-x_L_ does not form fibers with humanin. These results support a general mechanism of sequestration of pro-apoptotic BCL-2 proteins into fibers by HN to inhibit MOMP.

The mitochondrial pathway of apoptosis is regulated by pro- and anti-apoptotic members of the B-cell lymphoma 2 (BCL-2) protein family (1). These proteins interact with each other to regulate mitochondrial outer membrane permeabilization (MOMP), a process in which the mitochondrial outer membrane (MOM) becomes porous and releases apoptosis-propagating molecules from the mitochondrial intermembrane space into the cytosol (2). MOMP is considered to be the final, irreversible trigger that fully commits a cell to apoptosis. Pores are formed in part by some of the pro-apoptotic BCL-2 proteins, and these proteins provide the physiological means for diffusion of large, death-propagating complexes from the intermembrane space into the cytosol (3, 4, 5). MOMP is deregulated in cancers as a mechanism to maintain cell immortality and is misregulated in other diseases to the effect of aberrant apoptosis (6). Furthermore, the potential for a cell to undergo incomplete MOMP, a process in which MOMP occurs, but a few mitochondria are unaffected, and the cell survives with DNA damage, was linked to nonuniform BCL-2 expression at mitochondrial membranes (7, 8). Apoptosis-activating molecules that inhibit anti-apoptotic BCL-2 activity or stimulate the pro-apoptotic proteins are desired for disease states in which apoptosis is down-regulated, as in cancers and incomplete MOMP. Apoptosis inhibitors that can stabilize the pro-apoptotic proteins in inactive forms are also highly sought after for treatment of diseases presenting up-regulated apoptosis activity, including neurodegenerative diseases and autoimmune disorders (9).

BCL-2 proteins share some common structural features, and the overlap of these features determines whether a family member is pro- or anti-apoptotic. These proteins can have up to four regions of sequence similarity called BCL-2 homology (BH) domains (BH1–BH4) and have similar α-helical globular folds: a single hydrophobic core helix surrounded by seven amphipathic helices. Some feature a ninth C-terminal helix that functions as a transmembrane domain (TMD) (10). The six anti-apoptotic family members: BCL-2, BCL-b, BCL-W, BCL-x_L_ (B-cell lymphoma–extra large), BFL-1, and MCL-2 contain all four BH domains. These bind directly to and inactivate the pro-apoptotic BCL-2 proteins or retrotranslocate them from the MOM to the cytosol as an active process (11, 12, 13). The pro-apoptotic proteins are more sequentially and structurally diverse. The pro-apoptotic BCL-2 members either have three BH domains (BH1–BH3) or a single BH3 domain, the BH3-only proteins. The three pro-apoptotic multi–BH-domain proteins: BAX (BCL-2–associated X apoptosis regulator), BAK, and BOK oligomerize at the MOM and directly induce pore formation, ultimately becoming part of the pore structures themselves (5, 14, 15, 16). These proteins are considered to be the effectors for mitochondrial apoptosis. BAX and BAK activities are regulated through interactions with other BCL-2 proteins, whereas BOK is constitutively active and primarily regulated through ubiquitylation pathways (17). The BH3-only family members are subclassified into apoptosis sensitizers or activators and are mostly disordered proteins in the cytosol. The five sensitizers, BAD, BIK, NOXA, BMF, and HRK, inhibit the anti-apoptotic BCL-2 proteins by competing with their binding sites for BAX and BAK (18, 19). This liberates the pro-apoptotic pore-forming proteins to translocate to the MOM. Conversely, the three activator BH3-only proteins: BIM, BID (BH3-interacting domain death agonist), and PUMA can directly interact with BAX and BAK, inducing conformational changes that culminate in MOM translocation and MOMP activation (20, 21). BID is the sole BH3-only protein that folds into a stable tertiary structure, adopting the same α-helical globular fold as the multi–BH-domain proteins but without conforming to the standard BH sequences. The multi–BH-domain BCL-2 proteins and BID are characterized by a hydrophobic groove that binds BH3 domains from other proteins, and these are the primary regulatory interactions between family members (22). Interactions between the BCL-2 proteins and their cofactors are carefully synchronized to maintain homeostasis while remaining primed to stimulate MOMP.

A class of mitochondrial retrograde signaling peptides called mitochondrial-derived peptides (MDPs) were observed endogenously interacting with BCL-2 proteins and affecting MOMP-mediated apoptosis (23, 24). MDPs are expressed from alternate open reading frames in regions of the mitochondrial genome that are normally transcribed for the two subunits of the mitochondrial ribosome. Many MDP sequences have identical copies in the nuclear genome (25, 26). These peptides have been described regulating apoptosis and age-related metabolism, as well as being potently cytoprotective against numerous stress factors (24, 27, 28). The founding member of the MDP family, Humanin (HN), was discovered nearly two decades ago in connection with Alzheimer's disease (AD) and has been the most extensively characterized (29). HN interacts with some of the pro-apoptotic BCL-2 proteins directly to affect MOMP and inhibits apoptosis under several death-inducing conditions (30). It is thought that the primary action of HN against the BCL-2 family is suppression of BAX MOM translocation and MOMP activation through several mechanisms that remain to be fully elucidated.

HN was shown to interact with BAX, BID, and BIM while limiting the release of apoptotic factors from the mitochondria (31, 32, 33). It forms specific complexes with BAX or BID in the cytosol that inhibit their MOM translocation (23). HN also disrupts the cytosolic oligomerization of BAX and BID and their associated MOM translocation. Interestingly, HN cannot remove the two pro-apoptotic proteins from the MOM once they are already there, but they can still form a complex in the MOM that prevents pore formation and recruitment of additional BAX, a process that is associated with uninhibited BAX/BID oligomerization (34). The interactions between BIM and HN have not been as well-characterized because of BIM's status as an intrinsically disordered protein. HN only interacts with one of the three BIM isoforms, the extra-long isoform (32).

Attempts to determine the structure of HN complexes with these proteins have been met with challenges because of their tendency to aggregate *in vitro*. There has been some success using mutated or truncated versions of HN that do not induce BCL-2 aggregation to gain some insight into the binding mechanisms of these interactions (23, 32, 35). Invariably, HN binding is associated with conformational changes in the BCL-2 proteins that culminate in their inactivation. We propose that the HN peptide's mechanism of inhibition against individual pro-apoptotic BCL-2 proteins may share some commonality. There is no degradation of the BCL-2 proteins associated with enhanced HN levels, so the inhibition mechanism must always involve sequestration of the pro-apoptotic proteins in a nondestructive way. Strong predilection to aggregation between the BCL-2 proteins and HN *in vitro* despite their well-documented *in vivo* interactions implies formation of biomolecular superstructures that functionally inhibit the pro-apoptotic proteins.

We recently reported a new mechanism for BAX sequestration and inactivation by HN from observing their mutual reformation into β-sheet fibers *in vitro* (36). Here, we present the progression of this investigation with characterization of β-sheet fibers resulting from the interactions of HN with BID. Using spectroscopic techniques, protein fragmentation with mass analysis, and EM, we show that BID and HN can reform into stable β-sheet structures and fibrillate. The fibers have a uniform diameter, and BID is incorporated into the fiber core as was observed with BAX; however, there are major structural variations between fibrillated forms of the two pro-apoptotic BCL-2 proteins. Fibrillated BAX was described with a small N-terminal portion exposed outside of the fiber while the rest of the protein refolded completely into β-sheets. Limited protease digestions of BID fibers revealed that alternating parts of the protein become more exposed or more protected. Two segments of BID are incorporated into the fiber, whereas other portions of the protein persist outside the fiber core. CD results suggest that these exposed lengths of BID may retain their α-helical secondary structure. We also observed the effects of HN mutations known to affect the peptide's cytoprotective and neuroprotective capacities. Mutating the sole cysteine at position eight of HN abrogates both total fibrillation and β-sheet formation. Finally, we attempted to gain insight into the binding mechanism by observing aggregation formation between HN and a model pro-apoptotic protein, B-cell lymphoma-extra-large (BCL-x_L_). Reacting HN with BCL-x_L_ produces more aggregates *versus* the pro-apoptotic proteins, but our data show a commitment of HN and BCL-x_L_ to form disordered aggregates rather than reformation into β-sheet fibers. Taken together, these results lead us to propose a novel BID rearrangement mechanism involving HN-induced destabilization of the protein and reformation into β-sheets with two segments incorporated into the fiber core. Although this structural transition is different from the BAX fibrillation with HN, our findings support pro-apoptotic BCL-2 sequestration into fibers with HN as a common mechanism for apoptosis inhibition. Further characterization of this BCL-2 protein fibrillation process will provide future inspiration for therapeutics designed to promote sequestration and inhibit apoptosis.

## Results

### HN induces BID conformational changes, and together they form fibers

**Figure 1:**
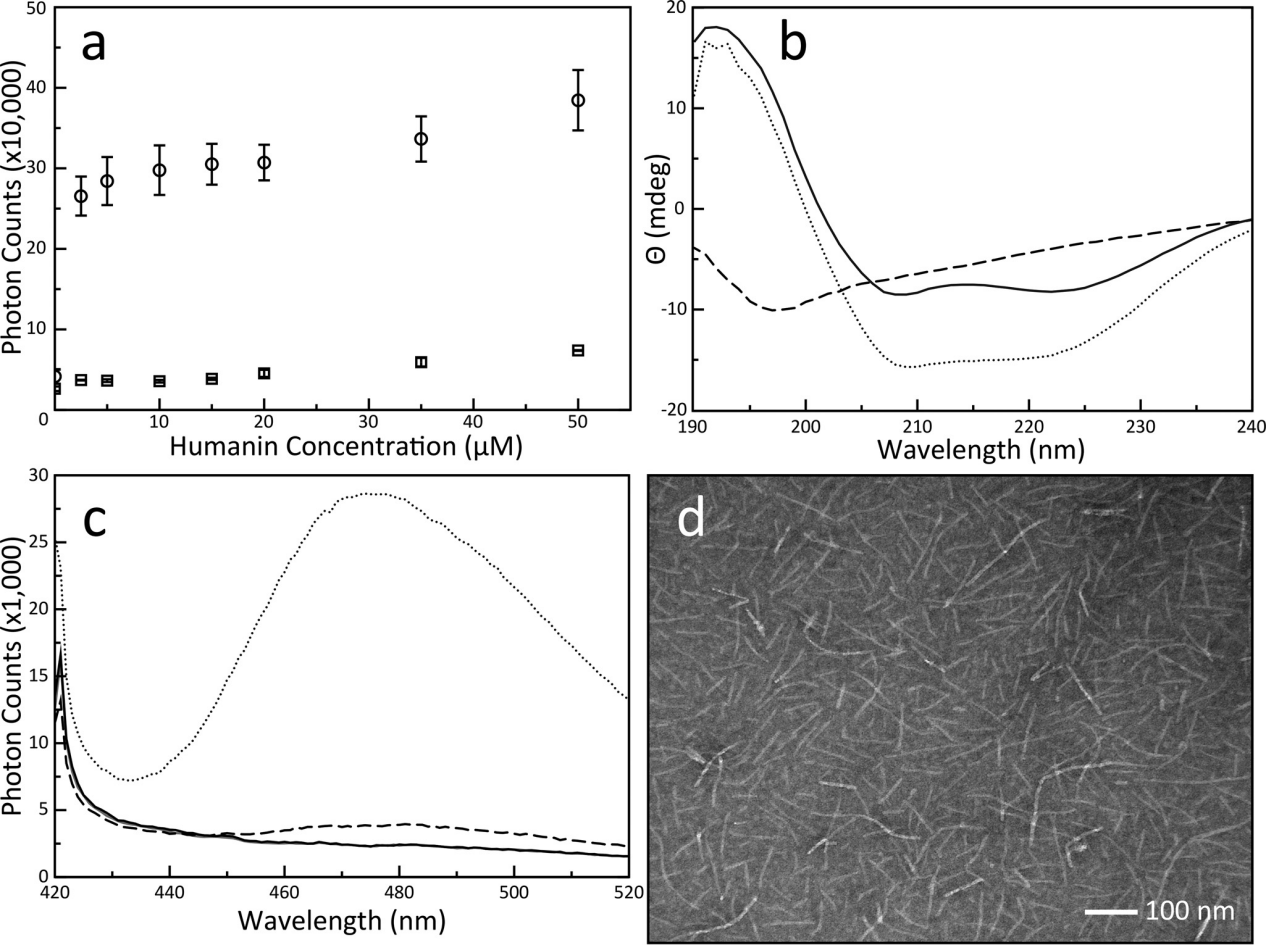
**Humanin induces conformational changes in BID, and together they form β-sheet fibers.***a*, increase in scattered photons from a 280-nm laser caused by aggregations in solution as a function of HN concentration in the presence (*circle*) or absence (*square*) of 5 μm BID. The *error bars* were calculated from the S.D. of three replicate titrations. Control titrations of buffer alone into 5 μm BID solutions did not show increased light scattering. *b*, CD spectra of 5 μm BID (*solid line*), 50 μm HN (*dashed line*), and a mixture of the two at the same concentrations (*dotted line*). The spectra were produced from three accumulated scans on a single sample. BID and HN alone produced spectra showing α-helices or disorder, respectively. Combining the two produced a spectrum with both α-helical and β-sheet properties, but there is no longer evidence of disorder, indicating that most of the HN has reformed into a stable structure. *c*, ThT emission spectra of the same solutions described in *b*. ThT did not interact with BID alone because the spectrum of the protein alone matched the buffer control, but ThT did have some activity with the peptide alone. When combined, the total fluorescence increased, and the maxima shifted by ∼7 nm, indicating formation of stable β-sheets. *d*, EM images of aggregations revealed fibers of uniform diameter and varying length.

Fibrillation propensity between BID and HN was initially probed using light-scattering spectroscopy and CD. These experiments confirmed that the two components, peptide and protein, are together required for significant aggregations to occur (Fig. 1*a* and Table S1). BID and HN are strongly reactive even at substoichiometric concentrations of the peptide. The titration shows that BID is initially more reactive for the fibrillation over BAX; however, the end points observed for titrations with either protein overlap (36). This indicates that the total aggregation propensity between BAX or BID with HN is constant at this 10:1 peptide-to-protein stoichiometry. There was some evidence for BID and HN conformational changes by CD (Fig. 1*b*). The spectrum of BID is typical of an all α-helical protein with two minima at 218 and 223 nm. The CD spectrum of HN has one minimum in the far-UV range indicating disorder. A mixture of the two produced a spectrum with mostly α-helical characteristics, but broadening over the 210–220-nm region implies that some β-sheets have formed.

β-Sheet formation was confirmed using thioflavin T (ThT)–binding experiments. The two reaction components alone and the mixture each produced three different ThT emission spectra (Fig. 1*c*). BID alone was indistinguishable from the buffer control, so the fully α-helical protein does not interact with ThT. The peptide alone induced ThT fluorescence with a broad emission maximum at ∼482 nm. This is consistent with some earlier investigations observing transient β-sheet structures in HN solutions under various conditions (37, 38). When HN was combined with BID, the total number of emitted photons significantly increased, and there was a 7-nm shift in the maximum of the emission spectrum to 475 nm. Considering that the CD spectrum of HN in our buffer showed a disordered peptide, these results together suggest that BID and HN combine to form a stabilized β-sheet conformation with greater affinity for ThT.

Finally, the reaction was observed by EM to reveal fibers (Fig. 1*d*). The fibers have a uniform morphology and mostly appear singularly, unassociated with other fibers. Notably, the networking observed with BAX and HN fibers were lacking in these BID fibers. BID fibers do not associate in parallel configurations and do not reach the extreme lengths observed in networked BAX fibers. Dilutions of the fibrillation reactions were imaged to obtain a data set of individual fibers. A sample of these is presented in Fig. S1. Individual fibers were outlined from the background after initial processing using a difference of a Gaussian band-pass filter (39). A total of 245 fibers were then characterized automatically using the ridge detection plugin for ImageJ/Fiji (40). This produced an average width and length for each fiber, which were binned to produce distribution histograms. The fibers have a narrow width distribution (Fig. S1e), and their length varies with an average of ∼50 nm and a maximum at ∼225 nm (Fig. S1f). The lack of networking observed in these BID fibers shows that their physical characteristics must deviate from the fibers produced using BAX.

To further characterize fibrillation sensitivity between BID and HN, some additional light-scattering titration experiments were conducted while varying conditions that are commonly known to amplify or trigger amyloid fibrillation (41). Enhanced reactivity was observed with higher temperatures (42), in the presence of detergent (43), and in buffers with increasing pH values (44) (Fig. S2). Reducing the temperature to 10 °C from 24 °C had no effect. However, increasing to 37 °C produced a significant 2.5-fold increase in the incidents of scattered photons (Fig. S2a). No light scattering above the baseline was observed in control titrations at 37 °C without BID. This indicates that the fibrillation mechanism depends on some specific interactions between BID and HN, because there is a reactivity enhancement at the higher temperature but no inhibition at the lower temperature. Simple particle collisions are insufficient to explain the fibrillation rate.

Even greater reactivity was observed in light-scattering titrations in the presence of a detergent. The nonionic detergent *n*-octyl-β-d-glucoside (OG) was selected for these experiments because of its established use as an activator of BAX/BID-mediated apoptosis and its common use as a reagent to solubilize membrane proteins (45). Furthermore, OG has been characterized as an accelerant for β-amyloid (Aβ) fibrillation (46). The highest concentration of OG tested was a 1% (w/v) solution, and this increased the incidents of light scattering ∼5-fold compared with control titrations without detergent (Fig. S2b). Titrations with only 0.25% (w/v) OG were also performed, but the curve for this more closely followed the 1% OG titration. A control titration of HN alone into 1% OG had a slightly higher baseline than usual, but there was no significant change in the incidents of scattered photons after the first titration point. This demonstrates that the detergent effects on fibrillation are nonlinear. OG exposes the hydrophobic portions of BID, and it may be these conformations that grant easier access to binding pockets which react with HN.

The largest increase in photon counts was due to raising pH values. For these experiments the reaction buffer was switched from 20 mm sodium acetate, pH 6.3, to 20 mm potassium phosphate buffers. This was to accommodate using pH values ranging from 5.5 to 8.0. Titration curves at pH values of 5.5 or 6.0 were nearly overlapped, and light-scattering values were in range of titrations conducted with the regular sodium acetate buffer. A significant increase in reactivity was observed at neutral pH with a 4-fold increase in scattered photons *versus* pH 6.0. This was pushed further at pH 8.0 with an ∼8-fold increase in light scattering (Fig. S2c). These observations are consistent with our hypothesis that HN targets the activated forms of pro-apoptotic BCL-2 proteins with more exposed hydrophobic binding sites. BAX and BID are suspected to undergo activating conformational changes during transitions from acidic to basic pH environments, so it was natural to expect that HN would be more reactive with BID as pH increases. It is also important to note that some light scattering was observed with a control experiment of HN titrated into the pH 8.0 buffer alone, but it is eight times smaller than the effect observed in the presence of BID. Less aggregation is detected at the beginning of the control titrations, but the end points are within error of those observed at pH 5.5 and 6.0. This is consistent with earlier reports of HN reforming into β-strands at neutral or higher pH values, and some aggregation from this phenomenon could be detected by light scattering (47, 48).

### BID is incorporated into the fiber structure

**Figure 2:**
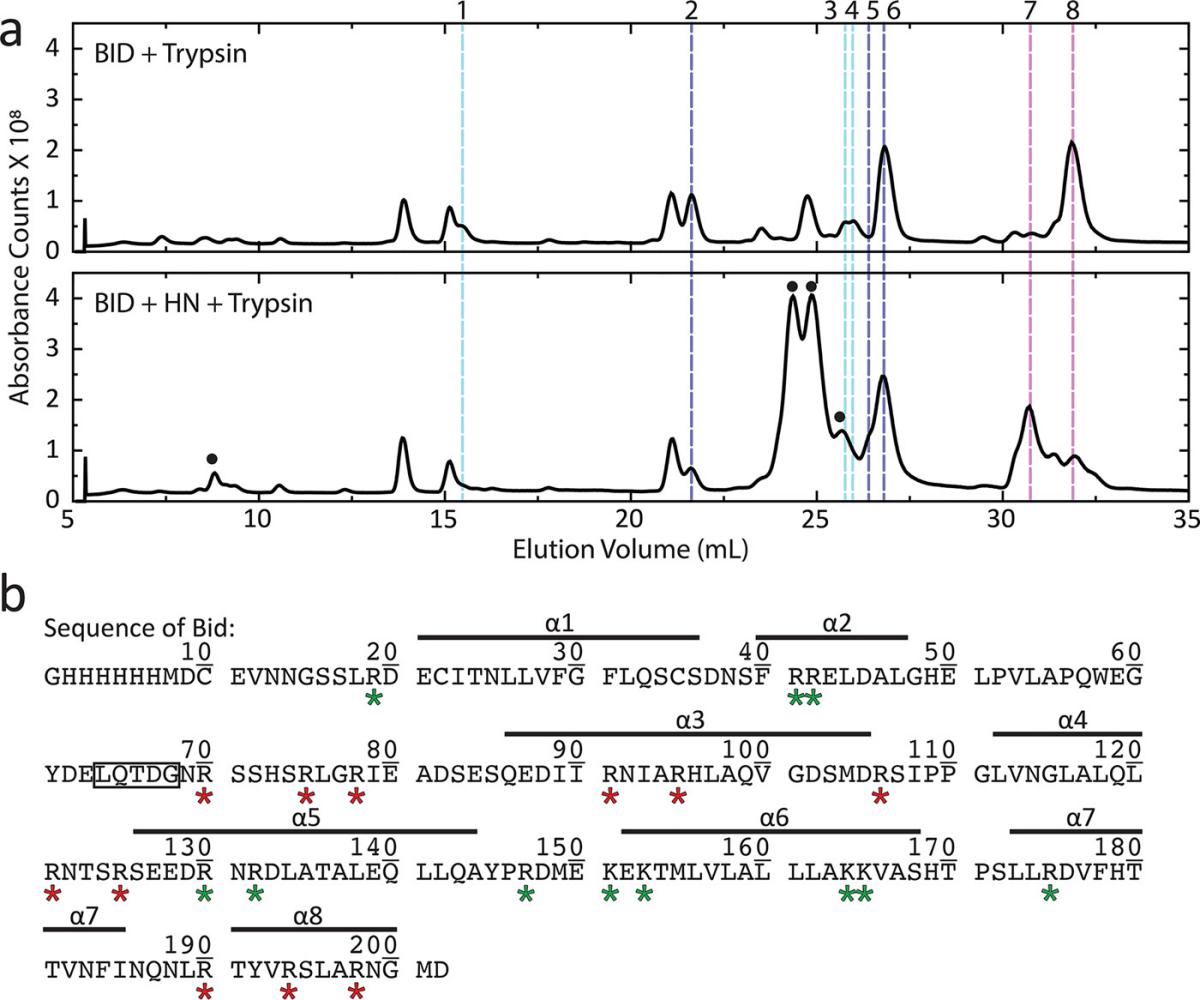
**BID is incorporated into the fiber and protected from proteolysis.***a*, total ion chromatograms of 2-h trypsin digestions with BID alone (*top panel*) and BID fibrillated with HN (*bottom panel*). Changes in the peak profile of BID digestion products are observed in the presence of HN. Differences in the relative intensities among three groups of peaks reveal that two regions of BID are incorporated into the fiber and are protected from proteolysis. *Magenta lines* highlight a long C-terminal fragment of BID, peak 8 (positions 79–202), that is more efficiently digested to a product corresponding to peak 7 (positions 126–190) when fibrillated. The peak 7 fragment spans three helices including the hydrophobic core helix α-6. *Cyan lines* highlight peaks 1, 3 and 4 corresponding to fragments 166–175, 133–147, and 154–164, respectively. The combination of these fragments is roughly the same sequence identified as the fragment from peak 7, which explains their attenuation in the fiber digestion. *Blue lines* highlight a second N-terminal region that is protected by fibrillation. Peaks 2, 5, and 6 correlating to BID fragments 43–70, 1–75, and 1–70, respectively, show that the cleavage sites in helix α-2 and a portion of the following loop region are protected from proteolysis. Peptides from HN cleavage products elute at 8.74, 24.35, 24.86, and 25.66 ml and are marked with *circles*. *b*, the sequence of BID with trypsin cleavage sites marked (*asterisks*) and α-helical segments highlighted. The caspase-8 cleavage sequence (63,64, 65, 66,67) is *boxed*. Some cleavage sites become more protected by the fibrillation (*green asterisks*), whereas others are more exposed (*red asterisks*).

We next sought to determine the regions of BID that are undergoing conformational changes and might be incorporated into the β-sheet structure. 2-h trypsin digestions were performed on fibrillated BID and the protein alone as a control. The digestions were quenched with acid, and fragment products were analyzed by LC–MS. These experiments revealed a unique digestion profile for BID when fibrillated with HN (Fig. 2*a*). The relative cleavage for each digestion site is affected by the fibrillation. Some sites become more exposed to trypsin, whereas others are more protected (Fig. 2*b*). This demonstrates that the final conformation of fibrillated BID varies significantly from its cytosolic structure. A summary of the complete deconvoluted MS data is presented in Table S2. Changes observed in three groups of peaks indicate two regions of BID that are incorporated into the fiber.

The first protected segment is shown by changes in peaks 2, 5, and 6 (highlighted by *blue lines*) correlating to BID fragments 43–70, 1–75, and 1–70, respectively. (Fig. 2*a*) The fiber digestion features reduction of peak 2 accompanied by the appearance of peak 5 as a shoulder of peak 6 (Fig. S3). Peak 6 also slightly increases in relative intensity. This shows that the cleavage sites at residues 41 and 42 are protected by fibrillation. Notably, the 1–75 fragment mass of peak 5 does not appear at all in the control spectrum, showing that the sequence surrounding residue 70 is potentially involved in the fibrillation process but remains exposed in the fiber. This is consistent with increased peak 6 intensity in the fiber sample, affirming that residue 70 was still 100% accessible to trypsin. This differential exposure site upon fibrillation is precisely in the middle of an extended loop region of BID. The appearance of peak 5 demonstrates that some portion of the unstructured loop is conformationally dynamic within the fiber structure and forming specific, transient interactions with HN, which exposes it for trypsin digestion. The N terminus of the unstructured loop is protected from proteolysis and incorporated into the fiber along with helices α-1 and α-2.

The second protected region is associated with changes in two groups of peaks. First are peaks 1, 3, and 4 (highlighted by *cyan lines*) corresponding to BID fragments 166–175, 133–147, and 154–164, respectively (Fig. 2*a*). The presence of all three of these fragments are reduced in the fiber digestions (Fig. S4), showing that the cleavage sites associated with these fragments are more protected when BID is fibrillated with HN. These fragments span the sequence of BID including the hydrophobic core helix, α-6, and portions of the preceding and following helices: α-5 and α-7, respectively.

Protection of the hydrophobic core is also observed from changes in the intensity distributions of peaks 7 and 8 (highlighted by *magenta lines*), fragments identified as residues 126–190 and 79–202, respectively (Fig. 2*a*). Peak 8 had a stronger signal in the control chromatogram because this large C-terminal segment of BID is mostly inaccessible to trypsin in the protein's compact α-helical conformation. The short digestion period did not result in a majority of the protein population becoming fragmented and unfolded to allow for more efficient cleavage. The peak profile for the fiber digestion shows peak 7 as a more common digestion product. Peak 7 is a subfragment of peak 8 spanning a region of BID including α-5 through α-7 (Fig. S5). This highlights that the entire hydrophobic core helix α-6 is incorporated into the fiber.

These observations are evidence that BID undergoes extensive structural rearrangements during the fibrillation and that its final conformation is more extended relative to the solution structure. Some portion of the C-terminal fragment identified from peak 8 is incorporated into the fiber and protected. The peak 7 fragment is a shorter digestion of the peak 8 fragment and closely matches the combined sequences associated with the three fragments identified by peaks 1, 3, and 4. This shows that those three peaks are attenuated because that entire segment of the hydrophobic core of BID spanning helices α-5 through α-7 is incorporated into the fiber structure and protected from proteolysis. The sequences flanking that region are in an extended conformation and exposed outside of the fiber core, which is the cause for more efficient trypsin digestion of the peak 8 fragment, whereas the peak 7 fragment is protected.

### Cleavage-activated BID segments can be fibrillated with varying strengths of incorporation

The final assays for determining if various parts of BID could be differentially incorporated into the fiber structure were inspired by the protein's regular biological function. The typical pathway by which BID is activated for pro-apoptotic activity is cleavage by the caspase-8 cysteine protease. BID is cleaved into two domains, p7 and p15, which remain associated with each other until the complex comes into contact with another BCL-2 protein, membrane, or other factor that stimulates their disassociation (49). The p15 domain, also known as truncated BID (tBID), has been described as the segment that ultimately activates BAX and takes part in the MOMP process (50). The p7 domain presently has no known function after it has been separated from tBID. Interestingly, the caspase-8 sequence (LQTDG) is immediately N-terminal to the protected cleavage site at residue 70 in the extended loop region between helices α2 and α3 of fibrillated BID. (Fig. 2*b*) This shows that the two segments incorporated into the fiber structure are uniquely from a portion of each caspase-8–cleaved BID domain. We wanted to test whether p7 or tBID incorporation depended on the other domain. For convenience, the caspase-8 cleavage sequence was mutated to a tobacco etch virus (TEV) protease sequence (ENLYFQG) (51). Using this construct (TEV-BID), we could produce cleaved BID with a tBID domain identical to the WT and a slightly modified p7 domain. Fibers produced with uncleaved and precleaved TEV-BID were identical to those produced using WT BID by EM (Fig. S6). Uncleaved TEV-BID was indistinguishable from WT BID by ThT fluorescence, whereas cleaved TEV-BID showed a 15% increase (Fig. S7). This observation implies that the two cleaved BID domains are involved in reactions with HN, and both are incorporated into the fiber. TEV cleavage of BID allows for a more efficient fibrillation, perhaps by easing the BID conformational transition, leading to higher ThT activity.

Overall fiber stability and TEV-BID dissociation were investigated by detergent solubilization. Aggregates produced from the fibrillation of cleaved TEV-BID and HN were submitted to multiple washes with a solution of 1% OG, a detergent known to induce dissociation of the p7 and tBID fragments (Fig. S8) (51, 52). Some of each cleaved domain was observed in the regular supernatant, representing a fraction of the aggregations that are not large enough to be pelleted by ultracentrifugation. The first OG wash contained a relatively larger fraction of the tBID domain. Most promisingly, successive washes and a final wash with distilled water did not solubilize additional TEV-BID domains or HN peptide monomers. This demonstrates both that the fibers are stable under mild denaturing conditions and that the cleaved BID domains can be incorporated into the fiber structure with p7 more tightly integrated *versus* tBID.

Finally, we tested the natural cleavage activity of WT BID fibrillated with HN using commercially sourced recombinant caspase-8. A sample of fibers was digested with 1 unit of caspase-8 for 6 h alongside a control of BID protein alone. It was observed that the cleavage rate of caspase-8 upon fibrillated BID is greatly accelerated (Fig. S9). Significant cleavage into the p7 and tBID subunits was observed in the fiber sample after only 1 h, whereas the control did not have obvious cleavage until the full 6 h had passed. This is in agreement with the trypsin digestion experiment, which suggested that the extended loop region containing the caspase-8 cleavage site might be conformationally dynamic within the fiber while remaining solvent-exposed. This also supports the assertion that BID is fibrillated in an extended conformation more readily cleavable relative to its solution structure.

### HN mutations affect fiber morphology

**Figure 3:**
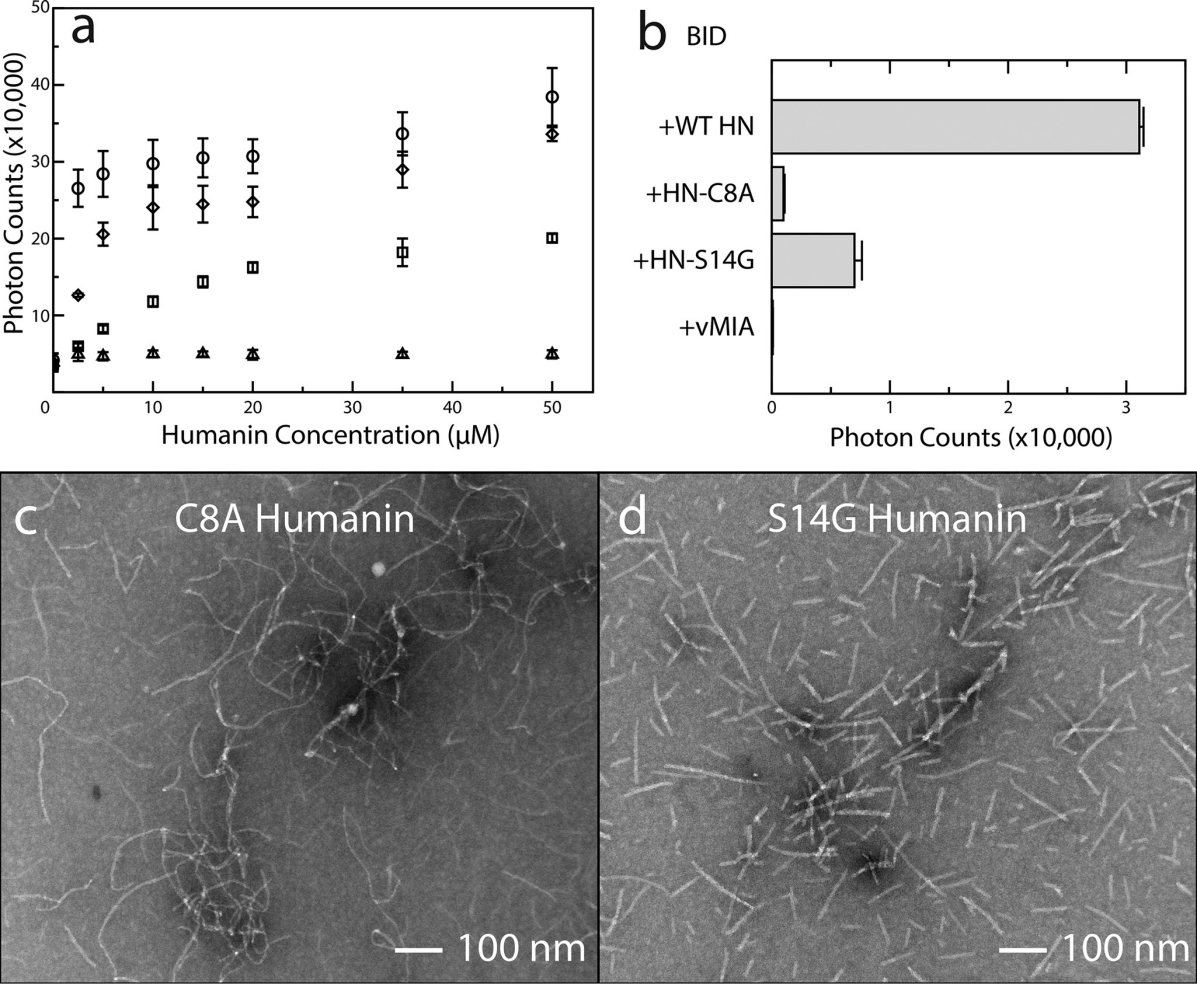
**Humanin mutants affect fiber morphology.***a*, light-scattering titration curves for 5 μm BID and each of the peptides. The WT HN/BID data (*circles*) are redisplayed as they appear in Fig. 1*A*. HN-C8A (*squares*) is less reactive with BID and does not form as many aggregates. HN-S14G (*diamonds*) aggregates less at the beginning of the titration but is within error of the WT HN values at the end. No increase in light scattering is observed with vMIA (*triangles*). The *error bars* were calculated from the S.D. of three replicate titrations. *b*, ThT fluorescence reported as the difference in signal intensity between solutions of 50 μm of each peptide with and without 5 μm of BAX. The HN peptides present some ThT affinity on their own but increase significantly when reacted with BID. Each fiber has a different ThT response, and the control peptide vMIA remains unchanged. The *error bars* were calculated from the S.D. of three replicate samples. c, EM image of fibers formed by HN C8A mutant and BID shows more irregular and thinner diameter fiber. *d*, EM image of HN S14G mutant and BID shows quite uniform diameter fibers that are shorter than WT HN and BID fibers.

Changes in fibrillation rates were observed when BID was reacted with some HN mutants (Fig. 3). The two mutants tested were selected for their physiological relevance. The C8A mutant (HN-C8A) has attenuated anti-apoptotic and neuroprotective activity. The S14G mutant (HN-S14G) is 1000-fold more active against apoptosis and neurodegeneration *versus* the WT peptide (29). A peptide derived from the BAX-interacting domain of a cytomegalovirus protein, viral mitochondria-localized inhibitor of apoptosis (vMIA), was utilized as a control peptide. Although the vMIA inhibition mechanism against BAX has been described structurally using this peptide, vMIA is also known to inhibit pro-apoptotic BID activity (53); thus its peptide serves as a reasonable control. Each of these peptides produced different curves over the course of a light-scattering titration (Fig. 3*a* and Table S3). HN-C8A displayed attenuated reactivity with BID. The number of scattered photons at the end point of the HN-C8A titration only reached half of the value observed with the WT peptide. This is consistent with observations that the C8A mutation is one of the factors that reduces affinity between HN and BAX. These data indicate C8 is important for the HN interaction with BID as well. HN-S14G does not aggregate with BID as readily as the WT peptide during the first half of the titration, but both curves reach essentially the same end points of their titrations. There was no increase in light scattering observed when BID was titrated with the vMIA peptide.

The rate changes associated with each mutant are accompanied by morphological changes in the fiber structure observed by ThT fluorescence experiments and EM. Each HN peptide displays different ThT reactivity with and without BID (Table S4). The HN peptides all have some baseline ThT affinity on their own. When mixed with BID, ThT binding increases significantly for the WT and S14G fibers, whereas HN-C8A barely increases at all (Fig. 3*b*). No increase in fluorescence is observed when BID is combined with vMIA peptide. These results confirm that BID reacts specifically with the HN mutants to form *bona fide* β-sheets with better affinity for ThT than the peptides alone. Lack of significant ThT fluorescence with HN-C8A combined with its attenuated light-scattering activity with BID implies that the mutant recombines with BID to form a stable structure, but very little of it is in β-sheet form. Indeed, EM of the aggregates produced by HN-C8A and BID revealed fibers with altered morphology *versus* the WT fiber (Fig. 3*c*). These fibers are thinner and have irregular kinks along their length. This suggests that the C8A mutant precludes the fiber from forming a repeatable tertiary structure that would result in more uniform fibers as seen with the WT peptide. The HN-S14G peptide also displayed attenuated ThT fluorescence with BID compared with the WT peptide, but this mutant's effect on fiber morphology is not immediately apparent (Fig. 3*d*). This mutant's fibers have approximately the same diameter as the WT fiber but are shorter on average. It is likely that the S14G mutant affects the fibrillation mechanism more than it does the final fiber structure.

### HN does not fibrillate with anti-apoptotic BCL-x_L_

To confirm that β-sheet fibrillation is something unique between HN and the pro-apoptotic BCL-2 proteins, BCL-x_L_ was used as a representative anti-apoptotic BCL-2 protein to assay its potential fibrillation activity with HN. BCL-x_L_ serves as an excellent anti-apoptotic comparison because both this protein and BID feature a long, unstructured loop region between helices α-2 and α-3. Furthermore, they share similar secondary and tertiary structures with exception of the BCL-x_L_ TMD (54, 55). The interaction between BCL-x_L_ and HN was first probed using light scattering as before. Titrating HN into a solution of 5 μm BCL-x_L_ produced an exponentially higher photon scattering count over the BID titration (data not shown). Additionally, the solution was notably turbid with a flocculent white precipitate that is characteristic of nonspecific protein aggregation. To verify that the increase in scattered photons was merely due to formation of large globular aggregates, these solutions were further analyzed by ThT fluorescence and CD.

**Figure 4:**
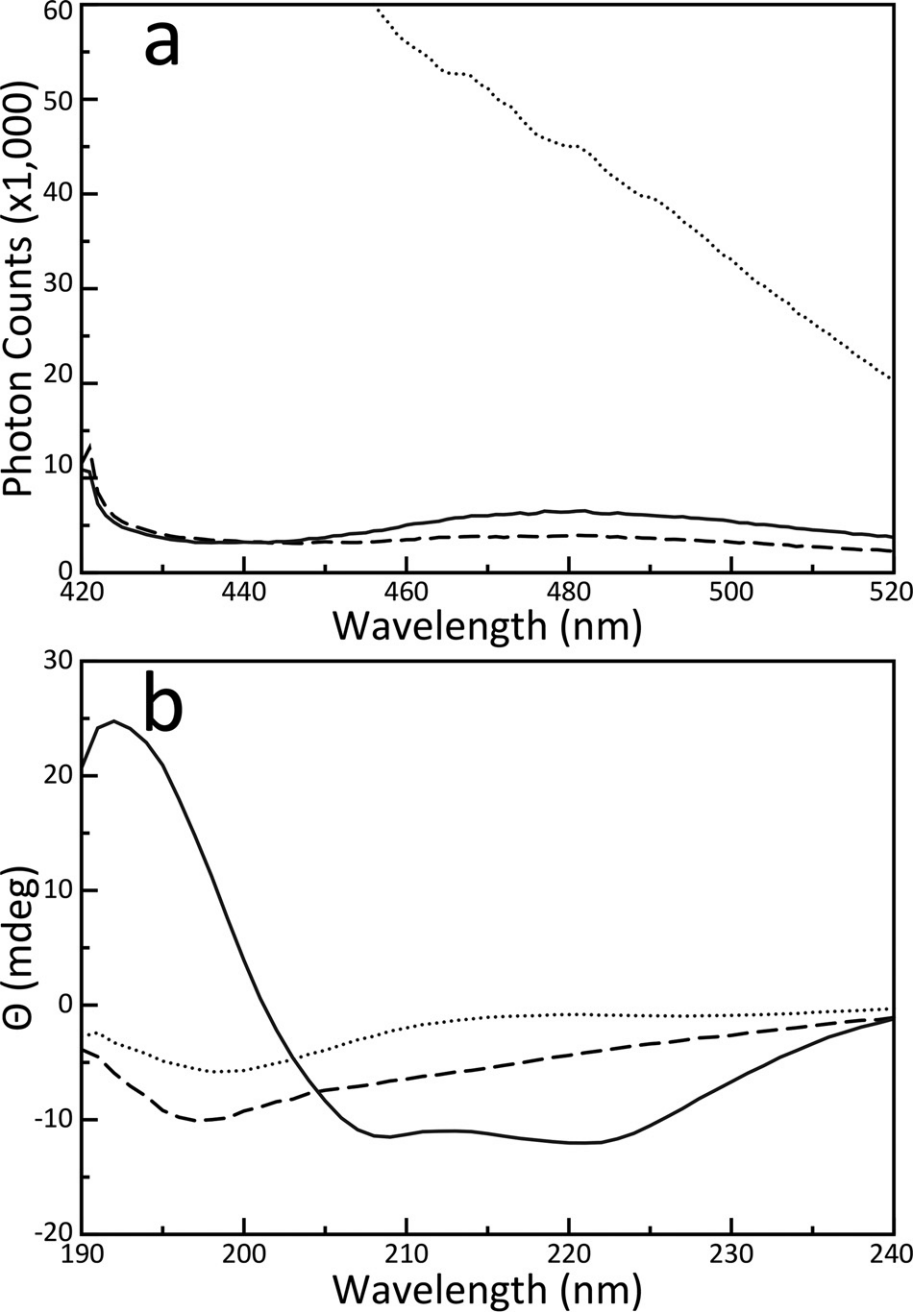
**Humanin does not form stable β-sheet structures with BCL-x_L_.***a*, ThT emission spectra of samples containing 5 μm of BCL-x_L_ (*solid lines*) and with 50 μm of HN added (*dotted lines*). The spectrum of 50 μm HN alone (*dashed lines*) is shown as a control. BCL-x_L_ alone shows some reactivity with ThT, but when combined with HN, the two aggregate so severely that the emission spectrum only shows scattering of the 410-nm excitation beam. *b*, CD spectra of the samples described above using the same key. Although the combination of BID and HN resulted in enhanced signal intensity and showed evidence of β-sheet formation, combination of BCL-x_L_ and HN reduced signal intensity relative to free BCL-x_L_ along with complete loss of BCL-x_L_ α-helical secondary structure. BCL-x_L_ and HN can only form disordered globular aggregates.

Large aggregations were immediately apparent in the ThT experiments (Fig. 4*a*). Interestingly, the solution containing only BCL-x_L_ had greater affinity for ThT over the HN peptide alone. It was earlier reported that BCL-x_L_ can reform into amyloid fibers under extreme temperature conditions; this observed ThT activity might reflect its propensity to form fiber aggregates (56). However, when BCL-x_L_ and HN are combined, the resulting emission spectrum is produced predominantly by light scattering of the 410-nm excitation beam because of relatively large particle formation. There is no apparent affinity for the aggregate with ThT. This was doubly confirmed with a CD spectrum of the mixture (Fig. 4*b*). The spectrum of BCL-x_L_ alone is characteristic of an all α-helical protein with two valleys nearly identical to BID. When combined with HN, the spectrum shifts to complete disorder. Where the combination of BID with HN resulted in enhanced signal intensity and broadening in the 205–225 nm range (evidence of β-sheet formation), BCL-x_L_ and HN instead show a reduction in signal intensity close to the noise over this range. Compared with the HN control, the mixture spectrum indicates that both molecules are fully committed to disordered aggregation with the only feature being a single valley in the disordered region at 198 nm. This indicates that the HN control has some transient β-sheet activity, which is abrogated by the addition of BCL-x_L_ because of the formation of disordered aggregates. Taken together, these results confirm that BCL-x_L_ and HN do not react specifically to form β-sheet fibers; which corroborates earlier investigations demonstrating that addition of HN to cells expressing BCL-x_L_ did not lead to a phenotype (23).

## Discussion

β-Sheet filaments associated with degenerative diseases are commonly referred to as amyloid fibers (57). Amyloid fibers are incredibly stable and represent some of the most toxic, degradation-resistant complexes known to biology (58). Early on, amyloid investigators believed aggregations and plaques formed by amyloids to be permanent structures, acting like “scars” for microscopic tissue damage. More recently a consensus has emerged that amyloids fibers can also undergo disaggregation mechanisms and that turnover of fibril formation is an active process in amyloid diseases (59). These observations lad to the hypothesis that amyloid aggregation/disaggregation processes might be purposeful and nontoxic in some biological pathways (60). Indeed, “functional amyloids” were soon discovered in organisms ranging from prokaryotes to *Homo sapiens* and are involved in diverse pathologies (61, 62, 63). Functional amyloids have been characterized that are involved in melanin biosynthesis (64), regulating necroptosis (65), storage of peptide hormones (66), forming antimicrobial membrane channels (67), facilitating the normal sperm maturation process (68), and responding to cell stress factors by stimulating mass amyloidogenic protein sequestration to induce a dormant cell state (69). We propose that the fibers formed between HN and the pro-apoptotic BCL-2 proteins follow a common theme of sequestration of a protein into a fiber as a cellular defense mechanism. The HN fibrillation against pro-apoptotic BCL-2 proteins functions as a fail-safe system to prevent MOMP when the cell is not ready for apoptosis.

The existence of a fibrillation system to regulate BCL-2–mediated MOMP is perhaps obvious in retrospect. HN was first recognized by Nishimoto and co-workers (29, 70) using a “disease-based death-trap” cDNA library constructed from amyloid-resistant occipital lobe tissue samples of a patient diagnosed post-mortem with AD. HN was found to inhibit neuronal cell death caused by the three types of familial AD genes or Aβ protein (29). HN interacts directly with Aβ and reduces its oligomerization while attenuating Aβ-induced neurotoxicity (71, 72). Neuronal cells affected by Aβ toxicity were shown to up-regulate expression of BAX and down-regulate expression of BCL-2 protein. This pathology was reversed by supplementation with HN (73). Conversely, some cancers were shown to up-regulate HN in response to apoptosis-inducing therapeutics (74). We propose that the mitochondria releases HN in response to these stress factors as a mechanism to affect the mass sequestration of pro-apoptotic BCL-2 proteins while disaggregating offensive intracellular amyloids. This pathology closely mimics the amyloid defense system bacteria employ against extracellular diphtheria or colicin protein toxins. The pore-forming domains of these toxins share homology with the BCL-2 family (75, 76). Many bacteria secrete peptides along with several other biomolecules that deploy into a dense network containing amyloid fibers described as a biofilm. The amyloidogenic biofilm serves as a scaffold for the bacterial colony to thrive on while it assists in evading host defense systems and neutralizing toxins that attack the colony (77). It stands to reason that the mitochondria, as a formally endosymbiotic organelle that was once a bacterium, would adapt this basic defense mechanism to regulate MOMP.

Here we have described a novel sequestration and inhibition mechanism for BID by fibrillation with HN. These results follow our earlier work detailing the fibrillation between BAX and HN; however, some subtle differences indicate that the two pro-apoptotic BCL-2 proteins have different fibrillation mechanisms. As before with BAX, the fibrillation process between HN and BID is swift and convoluted. It involves significant structural rearrangement of α-helical BID to a conformation with mixed secondary structure, accompanied by conversion of HN from disorder to order. The major difference is that characterization of BAX fibers suggested nearly complete conversion of the pro-apoptotic protein to β-sheets, whereas the BID fibril superstructure appears to be comprised of β-sheets and some residual helices. The helical structure is likely from segments of BID that are not incorporated into the fiber structure, whereas a majority of the BAX sequence is directly assimilated. The primary evidence for mechanistic differences comes from variability in the apparent rate of aggregation formation and trypsin fragmentation of BAX *versus* BID fibrils.

By light scattering BID is more reactive with the peptide over WT BAX. BID formed aggregations more readily with HN with the total count of scattered photons reaching a plateau at a ratio of 2:1 protein to peptide. BAX also reacted nonlinearly, but the increase in scattered photons never plateaued over the course of the titration. However, both reactions seem to reach the same end point. Interestingly, the BID titration more closely follows the curve observed using a BAX mutant featuring truncation of the C-terminal TMD. This BAX mutant better resembles BID in tertiary structure, with no autoinhibition of the BH3-binding groove by occupation of this site with a TMD. This is evidence further supporting the observation that HN is primarily engaging with globular BID at its BH3-binding groove.

This binding mode was first proposed in some elegant work by Marassi and co-workers (35), who resolved a NMR structure of BID complexed with a truncated form of HN shown to retain some of its anti-apoptotic activity while reducing aggregation propensity with BID. This HN peptide gained an α-helical secondary structure when bound with BID, and the interaction site was predicted to be the protein's BH3-binding groove. We propose that this previously determined model represents an important initial complex between BID and WT HN that goes on to induce significant conformational rearrangement of both molecules as part of the fibrillation mechanism. We hypothesize that the WT HN peptide stimulates destabilization of BID, which results in reformation of the two molecules into β-sheet fibers.

Our light-scattering experiments under varying conditions illustrate the sensitivity of the rate of fiber formation to environmental conditions. This suggests the possibility of BID and HN interaction that promotes fiber formation to be modulated by intracellular pH, temperature, and membrane localization.

**Figure 5:**
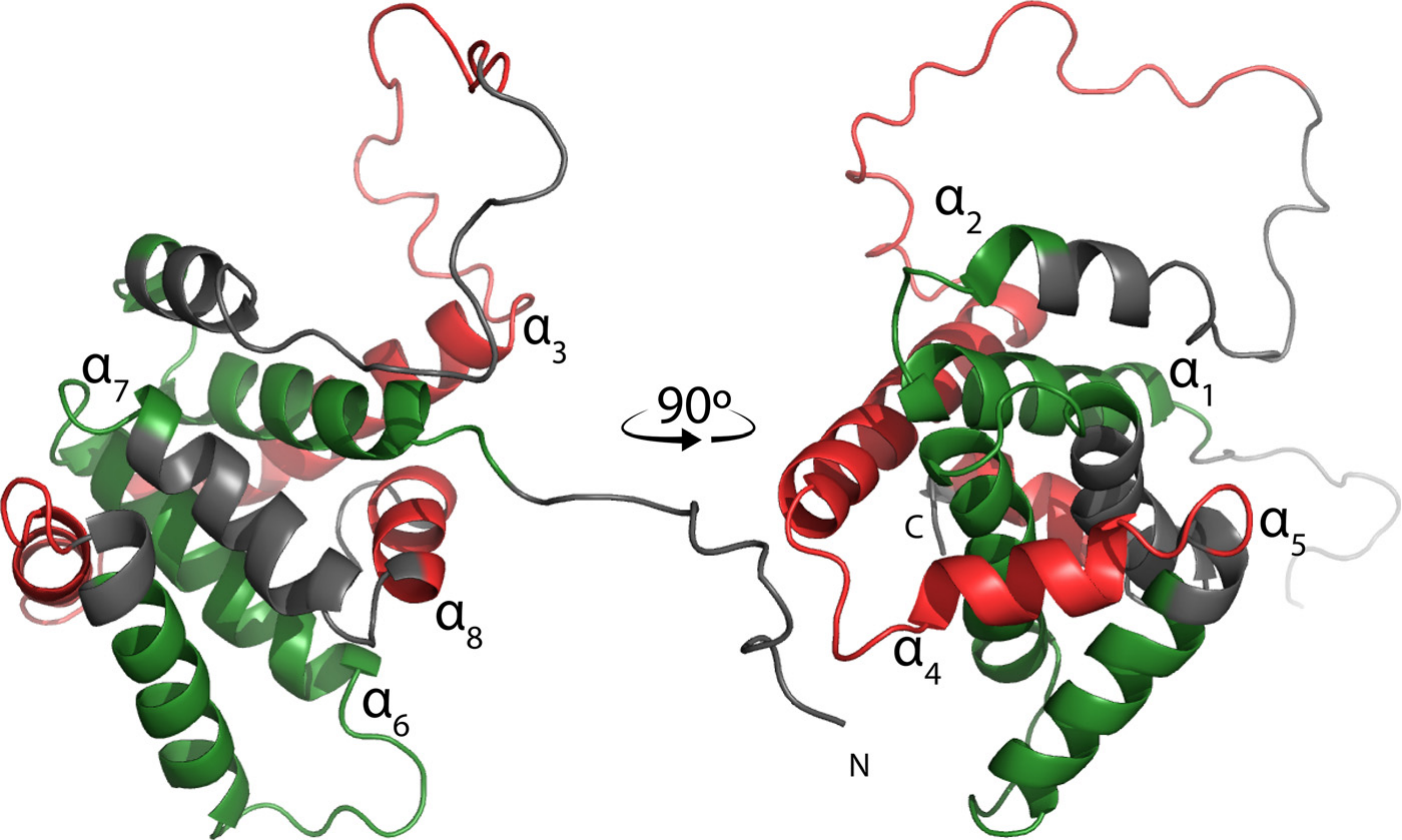
**Protected and deprotected regions of fibrillated BID.** The cytosolic structure of human BID (Protein Data Bank code 2BID) highlighting regions of the protein that are protected (*green*) and deprotected (*red*) by fibrillation with HN. Helices are labeled at their N-terminal ends. A region of the p7 segment containing the caspase-8 cleavage sequence in an unstructured loop region is not protected. Also, from the p15/tBID segment, the hydrophobic core helix α6 and part of the two flanking helices comprise the largest protected segment. Parts of the tBID segment immediately N- and C-terminal to the protected region become more solvent-exposed, indicating that fibrillated BID features an extended conformation relative to the cytosolic structure. Regions colored in *gray* represent stretches of the amino acid sequence that transition from fiber-incorporated to solvent-exposed.

Understanding why BID would have some residual α-helical structure even when fibrillated requires considering how this protein is regulated *in vivo*. BID is activated by cleavage into two segments, p7 and p15, by the apoptosis signal transducer caspase-8. The p15 segment, also known as tBID, goes on to interact with and activate BAX to induce MOMP. The p7 segment alone currently has no known function. Our trypsin digestions show that two regions of BID, one from each cleaved segment, are incorporated into the fiber and protected from proteolysis (Fig. 5). Interestingly, the caspase-8 cleavage sequence is immediately N-terminal to the trypsin cleavage site at residue 70 within the unstructured loop region. The other protected region is part of the cleaved p15 fragment and contains the hydrophobic core helix α-6 along with some of the two neighboring helices. Sequences immediately flanking the protected segment then become deprotected, showing that the protein is in an extended conformation within the fiber. Our data suggest that regions associated with α-helices 3, 4, and 8 are not incorporated into the fiber structure and remain solvent-exposed. These regions may retain their α-helical secondary structure even when fibrillated, which could explain why there is still an α-helical signature in the fiber CD spectrum.

Additional structural analysis is required to describe the true nature of the caspase-8 site when fibrillated. Given that precleaved TEV-BID is slightly more reactive than WT BID by fluorescence and that the caspase-8 cleavage site remains cleavable in fiber-incorporated BID might indicate that releasing conformational strain by cleavage helps to promote fiber formation. This can have an added inhibitory effect by more efficiently sequestering activated BID by caspace-8 into more energetically favorable β-sheet fiber conformations and blocking activation of downstream apoptosis factors.

Fiber formation with TEV-BID highlights that HN reacts with both domains, and our data hinted that the p7 domain might have a tighter integration into the fiber *versus* tBID. Normal fibrillation with precleaved TEV-BID indicates that covalent linkage of the two domains are not required for the mechanism to proceed. HN triggers a conformational rearrangement that involves both BID domains, whereas the α-helical globular protein mutually refolds into β-sheets along with the peptide. Results from the OG resolubilization experiment (Fig. S8) highlight that the fiber-incorporated conformation of the full-length protein is conserved in precleaved, fibrillated TEV-BID. A small fraction of the total amount of tBID was detected in the initial detergent wash, whereas almost no p7 was observed. This observation corroborates with the trypsin digestion when one considers the three exposed α-helices. All three belong to the tBID domain, meaning tBID is more exposed to OG over p7, and the detergent can more effectively destabilize tBID. Furthermore, precleaving the extended loop might reduce some conformational strain, which results in a more readily converted β-sheet structure (Fig. S7), but the basic conformation of the fibrillated BID domains are retained.

Fiber formation among the mutant HN peptides and lack of fibrillation when HN was combined with BCL-x_L_ imply a common inhibition mechanism for HN against the pro-apoptotic BCL-2 proteins. Disparity between the ThT fluorescence values for WT HN and HN-S14G fibers despite their similar light-scattering titration profiles implies that the final β-sheet structure of the HN-S14G mutant deviates from the WT fiber. Furthermore, the inability of the HN-C8A fiber to increase ThT affinity shows that these fibers lack significant β-sheet structure. Taken together, these observations have some important implications for the peptide's mechanism *in vivo*. It is likely that β-sheet formation is critically important for HN activity against pro-apoptotic BCL-2 proteins. Lack of β-sheet formation when HN was combined with BCL-x_L_ confirms that anti-apoptotic BCL-2 proteins do not react specifically with HN to affect MOMP pathology. This is evidence of a common inhibition process by HN against the pore-forming BCL-2 proteins through the presently described fibrillation mechanism.

In conclusion, we have detailed the inhibition and sequestering of BID into amyloid-like fibers along with HN. This is a nondestructive mechanism that prevents BID from being activated and inducing MOMP along with BAX. Sequestration of the pro-apoptotic BCL-2 proteins into fibers can potentially allow cells to respond dynamically to death signals. We propose that this fibrillation process is a general trend between HN and the pro-apoptotic BCL-2 proteins it has been shown to interact with. This mechanism represents a novel type of functional fiber that regulates cell death along with the BCL-2 proteins. Continued investigation into the fibrillation process and structurally validating sequestered conformations of pro-apoptotic BCL-2 proteins could prove to be fruitful toward the development of therapeutics targeting them.

## Experimental procedures

### General procedures

Experimental protocols for fiber characterization using light-scattering spectroscopy, ThT fluorescence, negative-stain EM, and trypsin digestions with HPLC–MS were performed as previously described (36). All chemical reagents were purchased from Sigma–Aldrich unless otherwise noted.

### BID and BCL-x_L_ expression and purification

Expression of N-terminal His_6_-tagged BID was performed as described previously (51). *Escherichia coli* cell pellets were resuspended and homogenized in His Buffer A (20 mm Tris-Cl, pH 8.0, 500 mm NaCl, and 5 mm imidazole) with Benzonase® nuclease (Merck) and cOmplete^TM^ protease inhibitor mixture (Roche). Insoluble material was pelleted by ultracentrifugation for 40 min at 185,500 × *g* and 4 °C. For the following chromatography purification steps, all flow rates were held constant at 5 ml/min. A 5-ml HisTrap HP column (GE Healthcare) was prepared by washing the resin with 5 column volumes (CV) of water and then equilibrating with 10 CV of buffer A. The supernatant was passed over the column, immobilizing the His-tagged BID. BID was eluted using His buffer B (20 mm Tris-Cl, pH 8.0, 500 mm NaCl, and 250 mm imidazole) with a gradient of 0–100% His buffer B over 20 CV. BID-containing fractions were pooled and buffer-exchanged into Q buffer (20 mm Tris, pH 8.0) using a PD-10 column (GE Healthcare). The protein was further purified by ion-exchange chromatography using a 5-ml HiTrap® 5 Q HP column (GE Healthcare) with a linear anion salt gradient up to 1 m NaCl over 20 CV. The BID-containing fractions were pooled, concentrated, and purified by size-exclusion chromatography (Sephadex G-75, GE Healthcare) using 20 mm sodium acetate, pH 6.3, 150 mm NaCl, 1 mm DTT, and 0.1 mm EDTA buffer. BID-containing fractions were pooled, concentrated, and exchanged into storage buffer (20 mm sodium acetate, pH 6.3, and 0.1% sodium azide) by filter centrifugation. Purified protein was stored at −20 or −80 °C.

TEV-BID was expressed and purified in the same way. The protein was cleaved by addition of 10 units of TEV protease during an overnight dialysis in TEV cleavage buffer (20 mm Tris, pH 8.0, and 2.0 mm DTT). The cleaved protein was collected and further purified by ion-exchange and size-exclusion chromatographies before being exchanged into storage buffer as described above. The above protocols for obtaining BID and TEV-BID consistently result in proteins with at least 95% purity as determined by LC–MS.

BCL-x_L_ was expressed in *E. coli* using a pTYB1 expression vector, similar to BAX recombinant expression. The BCL-x_L_ expression and purification protocols follow those previously reported for BAX (Protein Data Bank code 1F16) (36).

### Fiber formation and purification

Solid HN peptides (Anaspec, Fremont, CA, USA) were predissolved in reaction buffer (20 mm sodium acetate, pH 6.3) to 1 mm stock. The purity of the peptide was confirmed to be at least 95% by LC–MS. Fibers were produced by preparing solutions of BID and HN in the reaction buffer. Fibers to be purified for EM were made by combining 5 μm BID with 50 μm HN in the reaction buffer in a volume of 100 μl and allowing the reaction to proceed at room temperature for 1 h. After the incubation period, fibers were pelleted by centrifugation for 15 min at 21,000 × *g* and room temperature. The pellets were further purified by successive washes in 1 ml of reaction buffer and then 1 ml of ultrapure water. Pelleted fibers were resuspended a final time in 100 μl of water. This final solution or the original supernatant from the reaction buffer were used to prepare samples for EM.

### CD

CD spectra were recorded on a Chirascan Q100 spectropolarimeter (Applied Photophysics, Leatherhead, Surrey, UK). The samples were measured in volumes of 200 μl with 5 μm BID and/or 50 μm HN using an autosampler with a 1-mm-path length Q100 Smart Cell (Applied Photophysics). Scans were collected from 240 to 190 nm with a 1-nm bandwidth using an integration rate of 1 s/point. Each experiment is reported as an average of three accumulated scans, and the spectra were baseline-corrected by subtraction of a spectrum collected on the reaction buffer.

### Detergent solubilization test

Fibrillation reactions were prepared using 25 μm BID and 100 μm HN in the reaction buffer with a volume of 100 μl. After fibrillation was allowed to proceed for 1 h, aggregates were collected by centrifugation at 21,000 × *g* for 15 min. The pellet was resuspended in 1 ml of 1.2% OG detergent solution (Affymetrix, Maumee, OH, USA), and the sample was allowed to equilibrate for 10 min before being recentrifuged. This detergent wash was repeated a total of three times. The pellet was resuspended and washed a final time with 1 ml of water before being resuspended a final time in 100 μl of water. Dilutions of each supernatant and the final pellet were analyzed by LC–MS as described above.

### Caspase-8 cleavage

Fibrillation reactions were prepared with 5 μm BID and 50 μm HN in 200 μl of reaction buffer. This was allowed to incubate at room temperature with gentle agitation for 1 h alongside a control reaction containing 5 μm BID only. Both were then supplemented with 1 mm DTT and 1 unit of recombinant caspase-8 (BioVision, Milpitas, CA, USA). 20-μl aliquots were taken at regular intervals, and the digestions were quenched by addition of 2 μl of 10% TFA. These were further diluted with 80 μl of MS solution (5% acetonitrile and 0.1% TFA). The samples were analyzed by LC–MS as described for trypsin digestions.

## Data availability

All data are contained within the article and the supporting information, except for light-scattering experiments with varying temperature, pH, and octyl glucoside concentrations. They are available upon request to the corresponding author.
