# Supplementary material for: Humanin selectively prevents the activation of pro-apoptotic protein BID by sequestering it into fibers
Source: J Biol Chem. 2025 Jan 13;295(52):18226–38. doi: 10.1074/jbc.RA120.013023 (PMC11843584; doi:10.1074/jbc.RA120.013023)
Supplement: Supplementary file 1 [file mmc1.docx]

**Supplementary Information**

Humanin selectively prevents the activation of pro-apoptotic protein BID by sequestering it into fibers

**Daniel L. Morris^1^, Sabrina Johnson^1^, Christopher K. E. Bleck^2^, Duck-Yeon Lee^3^, and Nico Tjandra^1^***

From the ^1^Laboratory of Molecular Biophysics, Biochemistry and Biophysics Center, National Heart Lung and Blood Institute, National Institutes of Health, 50 South Drive, Bethesda MD, 20814; ^2^Electron Microscopy Core Facility, National Heart Lung and Blood Institute, National Institutes of Health, 14 Service Road West, Bethesda MD, 20892; ^3^Biochemistry Core Facility, National Heart Lung and Blood Institute, National Institutes of Health, 50 South Drive, Bethesda MD, 20814

Running title: BID sequestration into fibers by humanin

*To whom correspondence should be addressed: Nico Tjandra: Laboratory of Molecular Biophysics, Biochemistry and Biophysics Center, National Heart, Lung, and Blood Institute, National Institutes of Health, 50 South Drive, Room 3503, Bethesda, MD 20892; tjandran@nhlbi.nih.gov; Tel. (301)402-3029; Fax. (301)402-3405.

**Keywords:** Apoptosis, BID, Humanin, Fibers, Electron Microscopy, Conformational Change, β-sheet, Amyloid


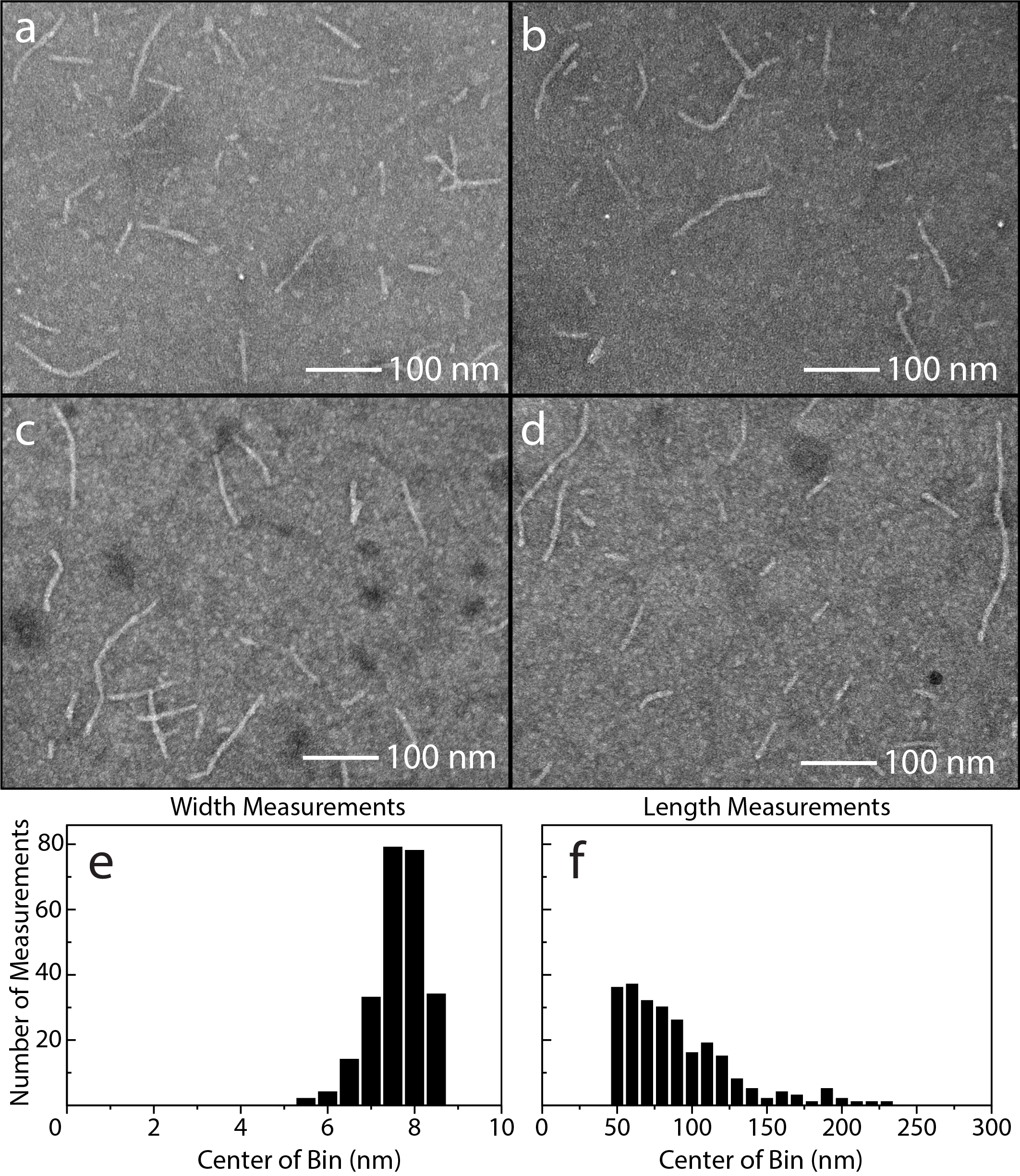


**Supplemental Figure 1 Statistical analysis of fiber width and length distribution.** *A-D*, Example images from the dataset of fibers collected at 40,000X magnification. A total of 245 fibers were processed for automatic length and average width detection. *E*, A histogram produced from the average width measurements of each fiber using a bin width of 0.5 nm. This plots a narrow width distribution around 7.5 nm. *F*, A histogram of the measured lengths shows an average length of about 50 nm with some fibers longer than 200 nm.


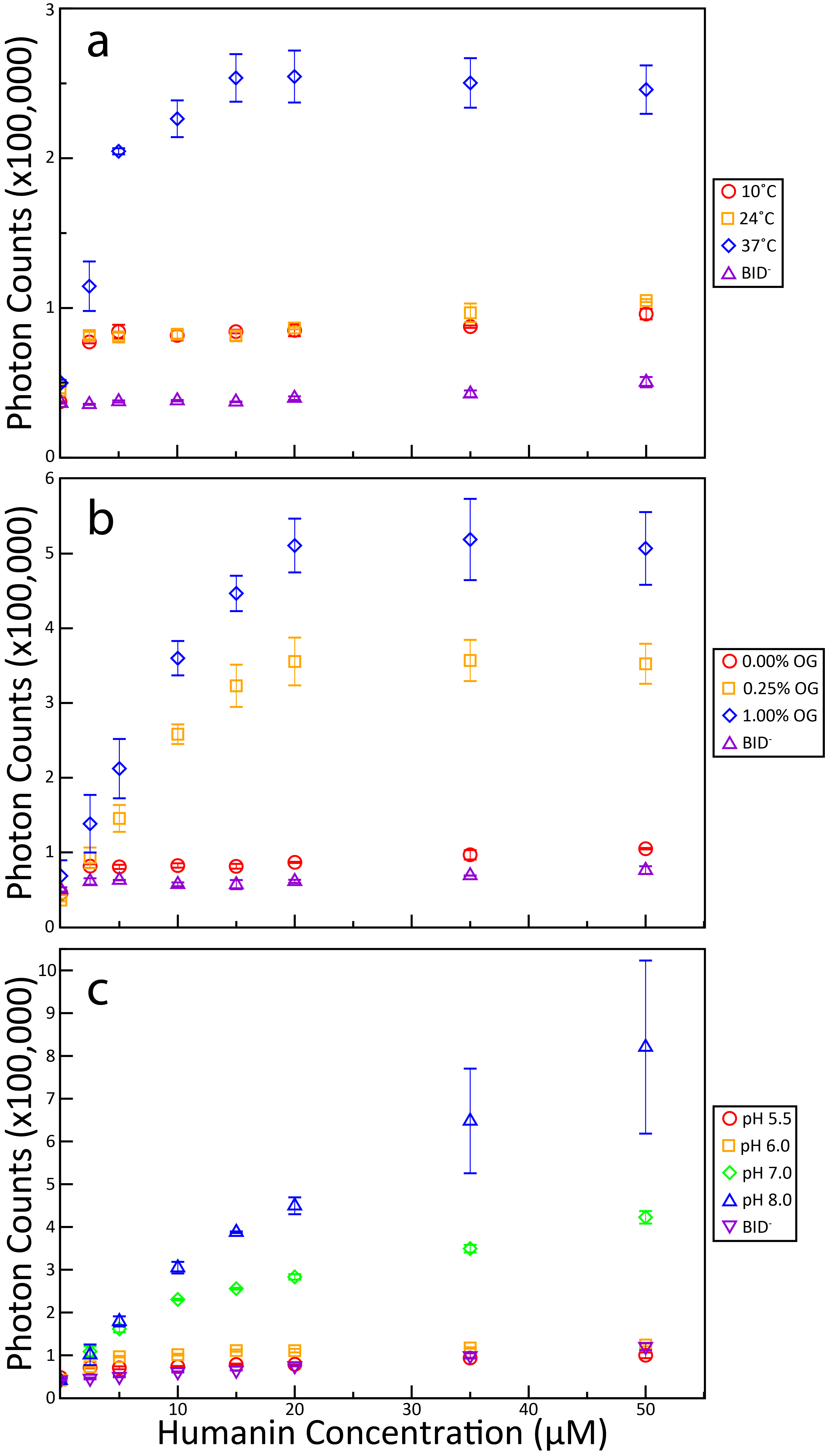


**Supplemental Figure 2 Light scattering titrations showing enhancement of BID and HN fibrillation in common amyloid-inducing conditions.** All curves were generated from duplicate titrations of samples starting with 5 μM of BID. *A*, Titrations are shown with increasing temperatures of 10°C (red circles), 24°C (orange squares), or 37°C (blue diamonds). No change in reactivity is observed at 10°C versus 24°C, but a significant increase is observed when the temperature is increased. A control titration of HN alone into buffer at 37°C (purple triangles) showed no activity. *B*, Titrations with increasing concentrations of OG detergent starting at 0% (red circle), stepping to 0.25% OG (orange squares), and finally reaching 1% OG (blue diamonds). A control titration of HN alone into 1% OG buffer showed no activity. The presence of even a small amount of detergent clearly shows a reactivity enhancement. *C*, The greatest reactivity enhancement was observed with increasing pH values. Very little change in reactivity was observed between titrations conduced at pH 5.5 (red circles) or pH 6.0 (orange squares), but reactivity increases significantly at neutral pH 7.0 (green diamonds) and even more so at pH 8.0 (blue diamonds). A control titration of HN alone into the pH 8.0 buffer indicates some homooligomerization of HN in more basic conditions.


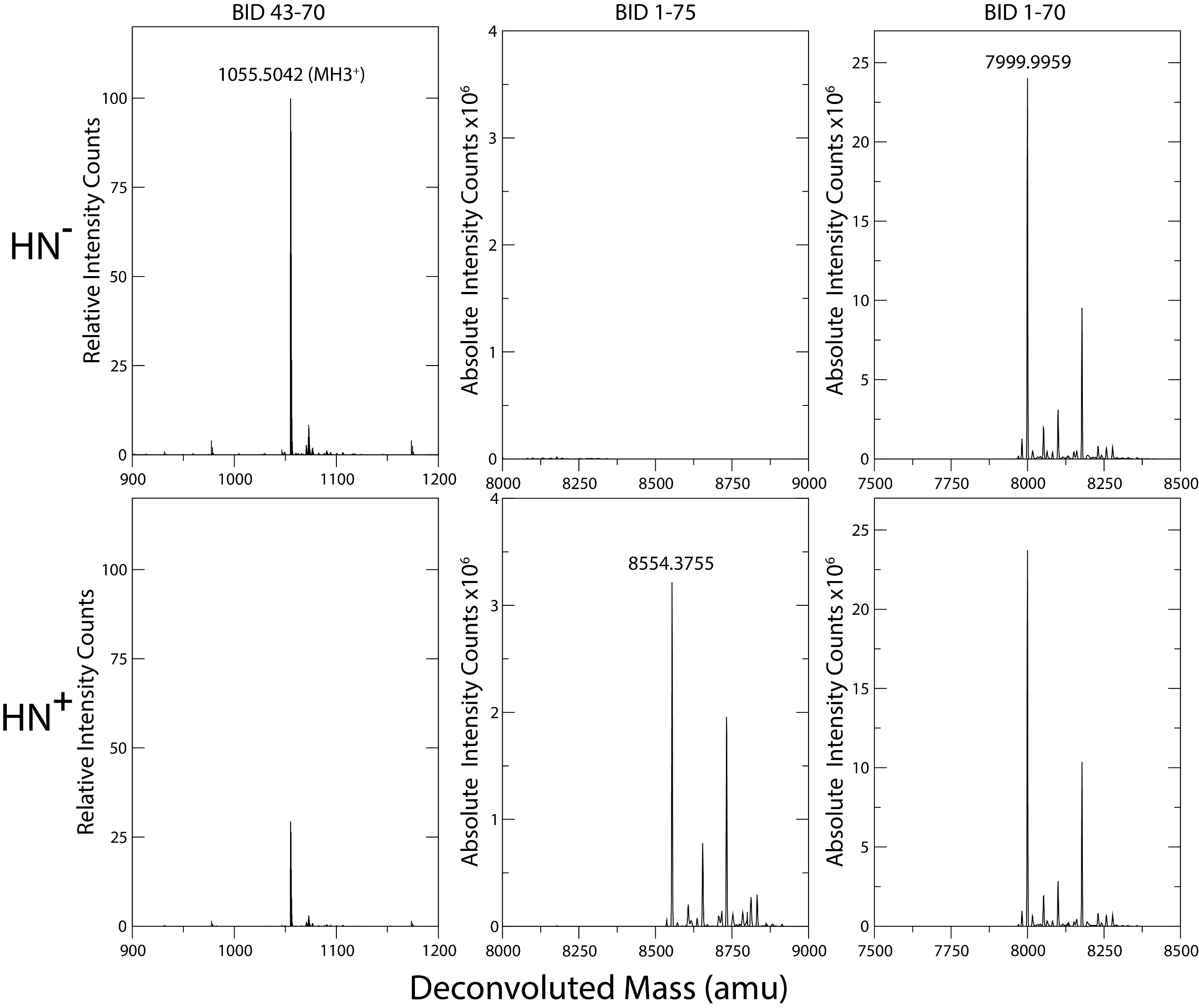


**Supplemental Figure 3 Deconvoluted Mass Spectra for peaks 2, 5, and 6.**


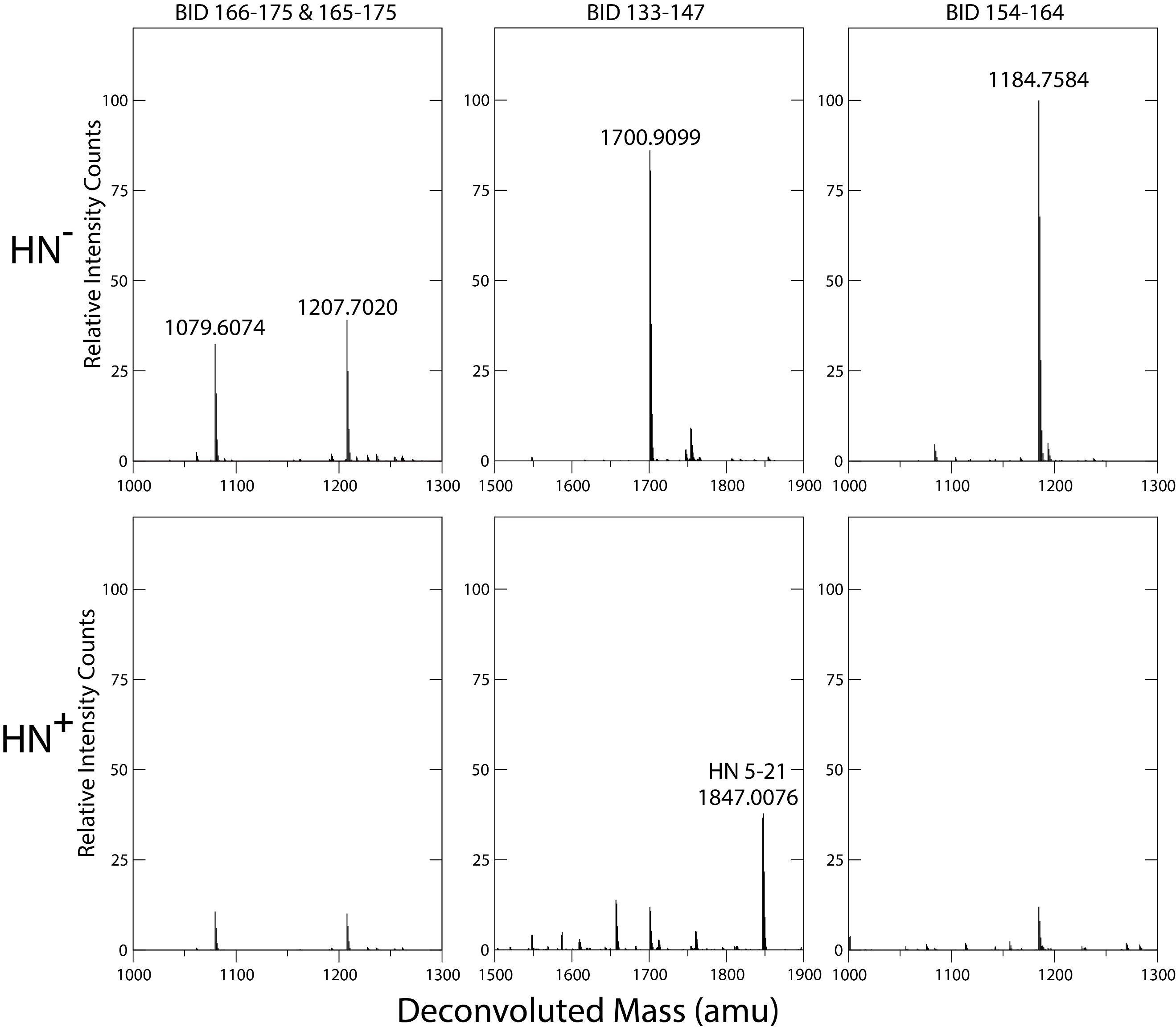


**Supplemental Figure 4 Deconvoluted Mass Spectra for peaks 1, 3, and 4.**


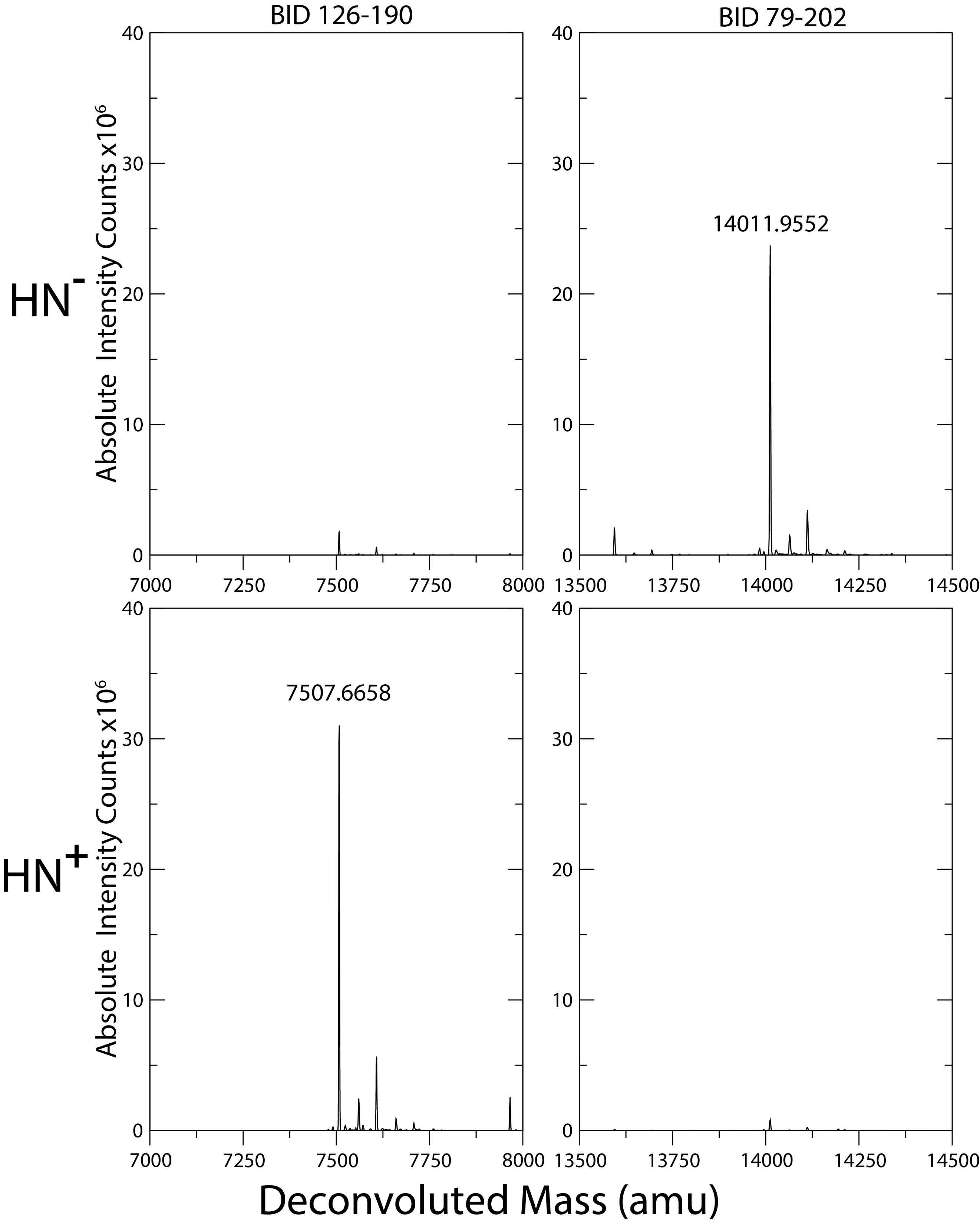


**Supplemental Figure 5 Deconvoluted Mass Spectra for peaks 7 and 8.**


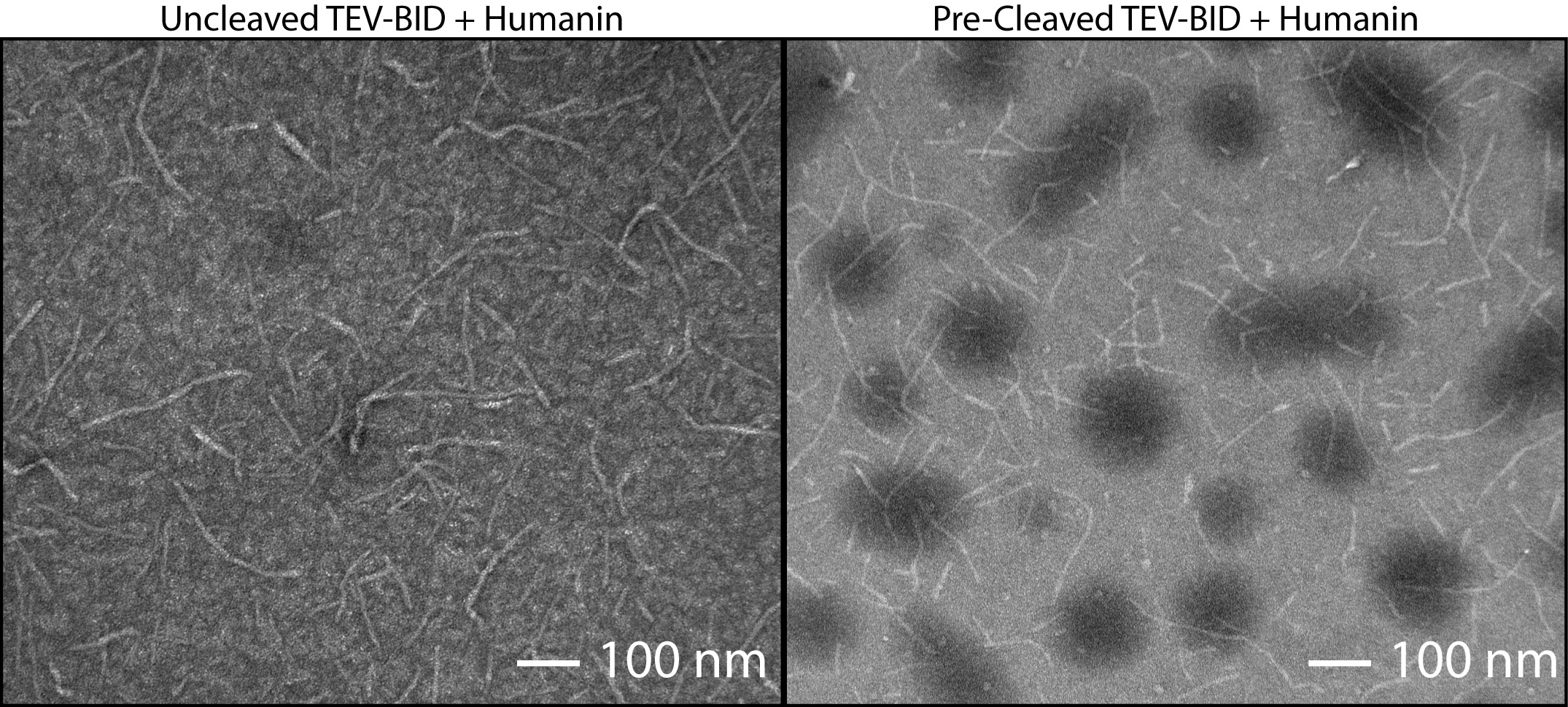


**Supplemental Figure 6 Examples of TEV-BID fibers imaged by negative staining.** Fibers produced by reacting uncleaved (left) and pre-cleaved (right) TEV-BID with HN are similar to the WT BID fibers.

**
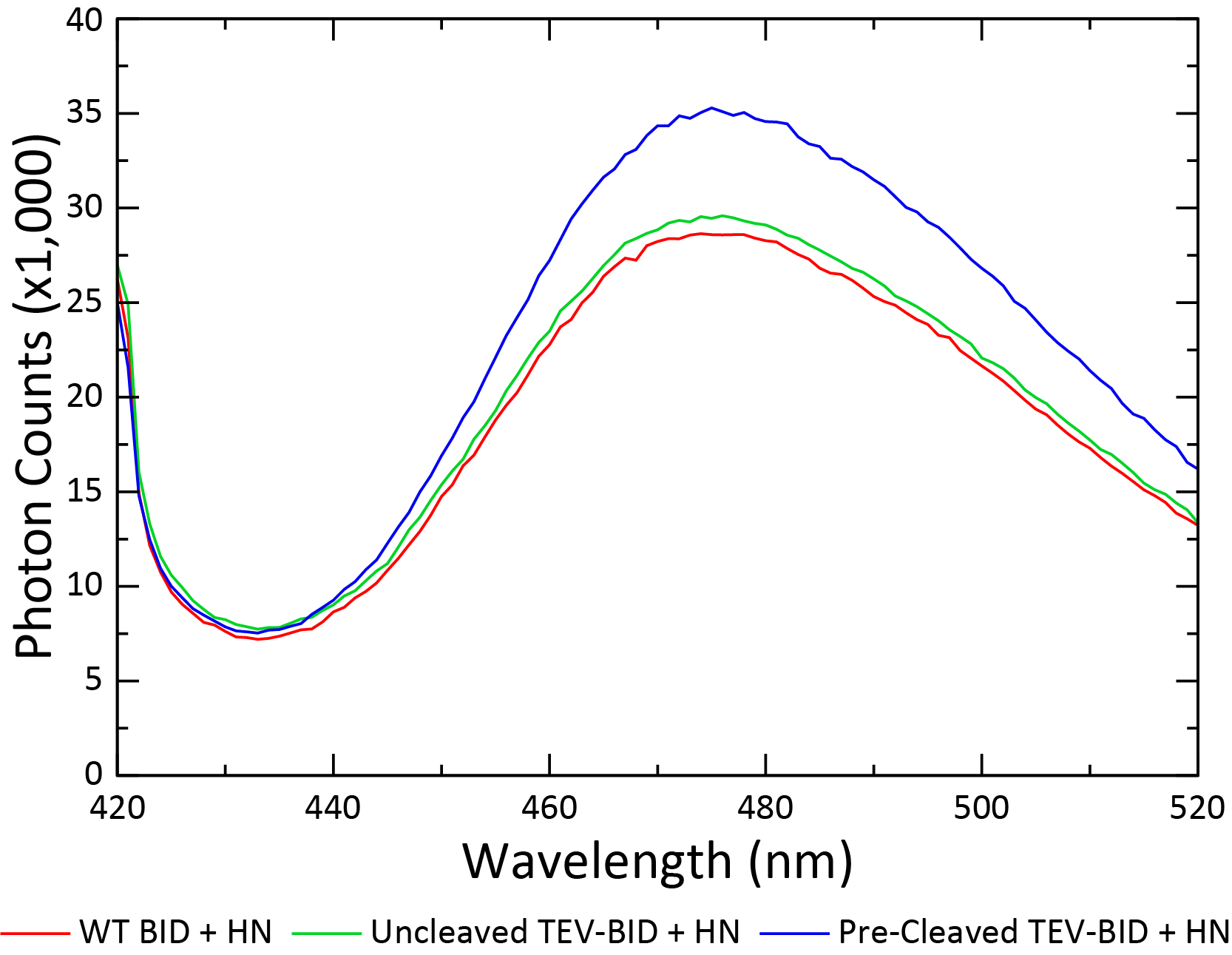
**

**Supplemental Figure 7 ThT binding and fluorescence with TEV-BID Fibers** ThT response for fibers made with 5 μM of each BID protein and 50 μM of HN. Uncleaved TEV-BID fibers show similar ThT binding activity versus the WT BID fibers. Pre-cleaved TEV-BID reacted with HN shows about 15% increased fluorescence over the full-length constructs. These curves were produced from the average of spectra collected on three different samples.

**
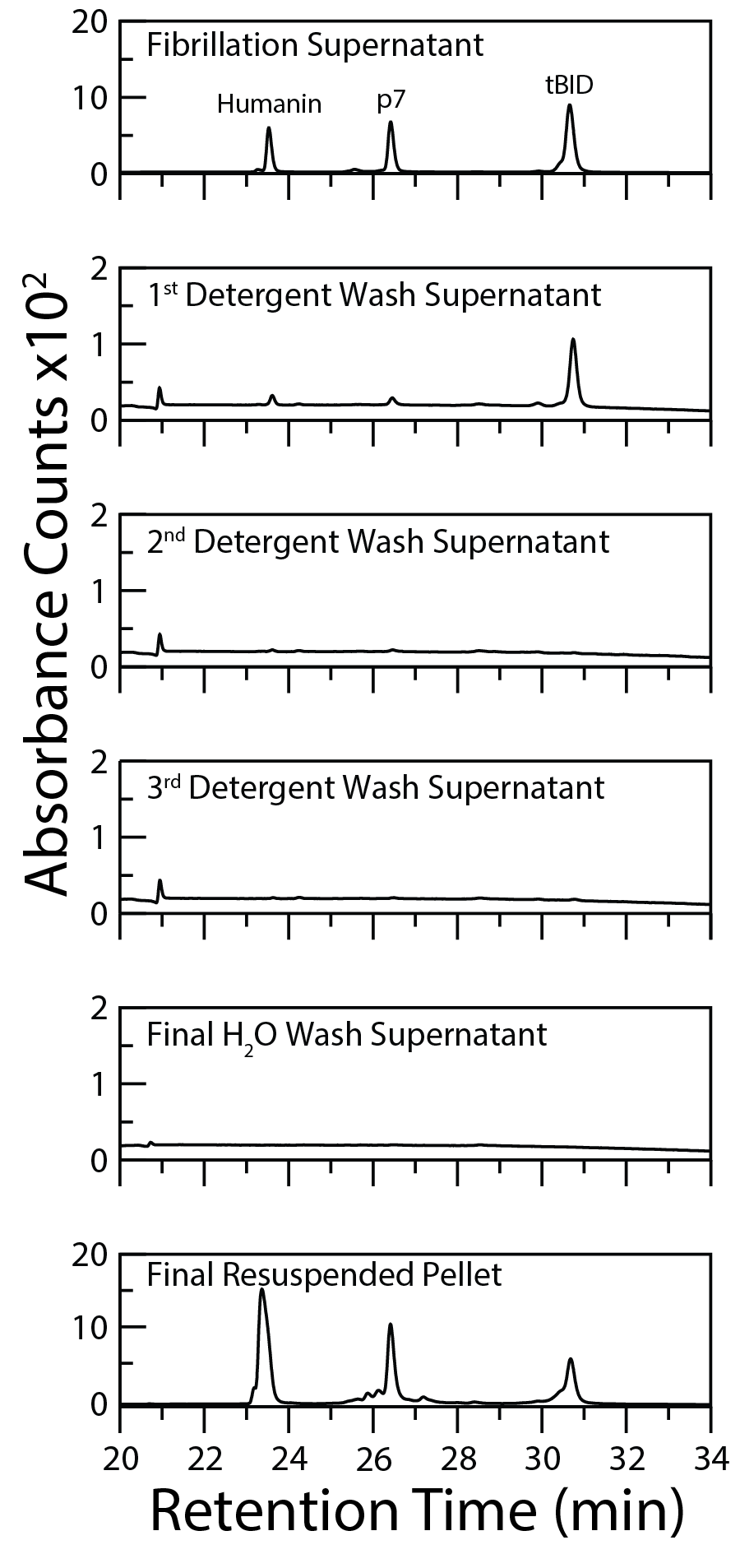
**

**Supplemental Figure 8 Detergent solubilization test.** Pre-cleaved TEV-BID was reacted with HN and fibers were washed by successive ultracentrifugation and resuspension of insoluble material into reaction buffer with 2% w/v OG detergent. Samples were analyzed by LC/MS to qualitatively determine relative incorporation of the p7 and tBID subunits. Some tBID is observed in the first wash, but successive washes do not further solubilize the fibers. The final pellet shows both cleaved domains with an apparent reduction in the ratio of tBID.


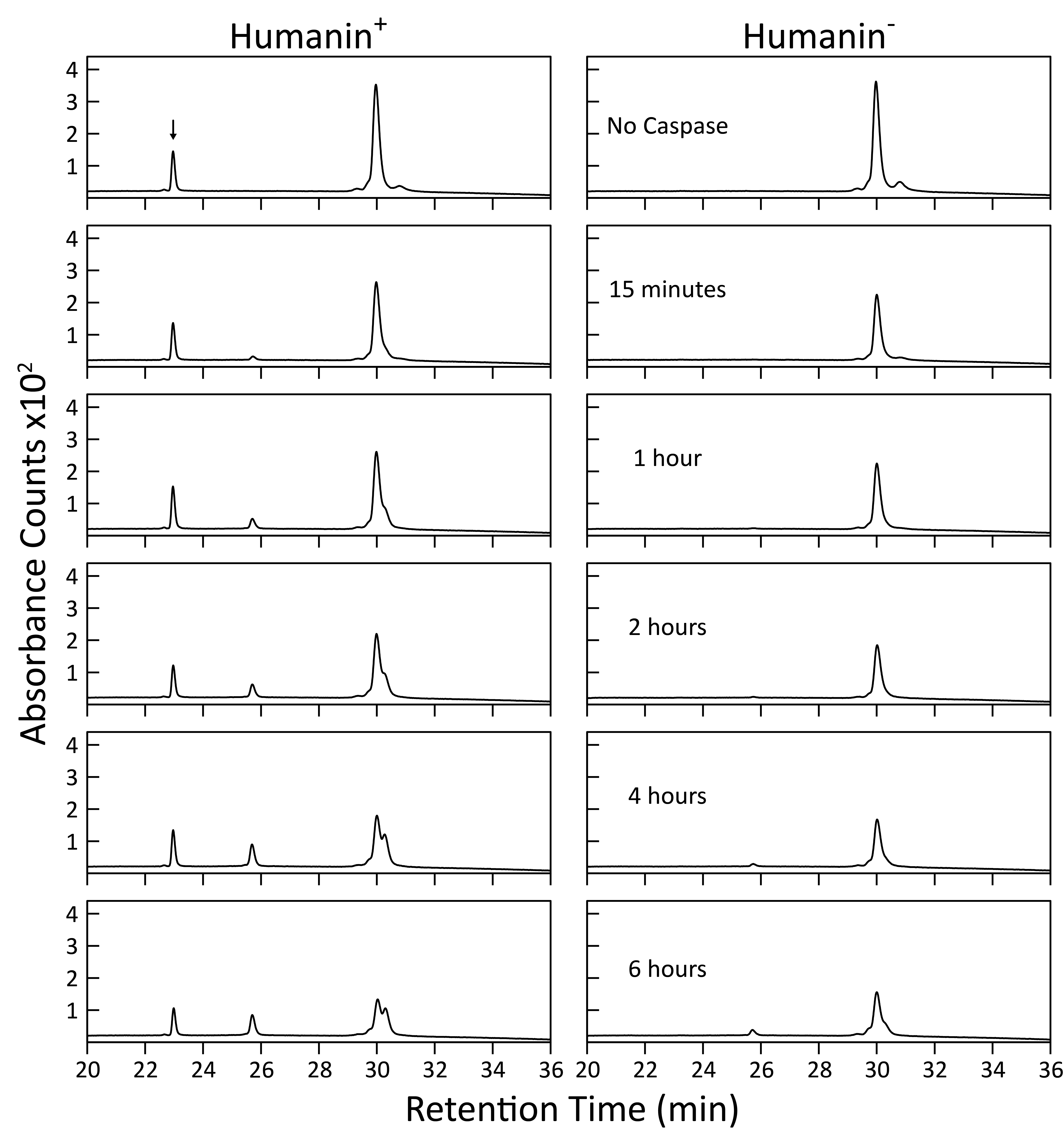


Supplemental Figure 9 The caspase-8 cleavage rate of BID is greatly enhanced when BID is fibrillated with HN. Samples of fibrillated BID (left) were simultaneously incubated with samples of BID alone (right) along with one unit of caspase-8 for a period of 6 hours. The samples were kept in a 37°C shaking incubator while taking aliquots for each time point. These were submitted to analysis by LC/MS to assay for generation of cleaved BID subunits. An arrow indicates the peak for HN in the fibrillated samples. Full-length BID elutes at ~30.0 min retention time. The p7 subunit elutes at ~25.8 mL while the tBID subunit elutes as a shoulder of the main full-length peak at ~30.2 mL. More efficient caspase-8 cleavage is clearly observed when BID is fibrillated with HN.

| **[Humanin] (μM)** | **5 μM BID #1** | **5 μM BID #2** | **5 μM BID #3** | **No BID #1** | **No BID #2** | **No BID #3** |
| --- | --- | --- | --- | --- | --- | --- |
| 0 | 4230 | 5234 | 3010 | 2231 | 2825 | 2825 |
| 2.5 | 25632 | 29872 | 24161 | 3641 | 3726 | 3744 |
| 5 | 25960 | 32611 | 26676 | 3393 | 3757 | 3760 |
| 10 | 28292 | 34054 | 26960 | 3344 | 3678 | 3678 |
| 15 | 28305 | 34056 | 29163 | 3676 | 3960 | 3963 |
| 20 | 28644 | 33781 | 29728 | 3915 | 4857 | 4853 |
| 35 | 30981 | 37528 | 32426 | 5173 | 6305 | 6301 |
| 50 | 35356 | 43731 | 36282 | 7320 | 7419 | 7410 |

**Table S1.** Raw photon count datapoints for the light scattering graph in Figure 1A.

| Retention Time (min) | Theoretical Value (m/z) | Observed Ion (m/z) | Peptide Sequence | Signal in Both Datasets? |
| --- | --- | --- | --- | --- |
| 6.32 | 435.1424 | 435.1425 | BID 199-202 | Yes |
| 7.39 | 904.3999 | 904.4009 | BID 126-132 | Yes |
| 8.44 | 778.3531 | 778.3525 | BID 148-153 | Yes |
| 8.74 | 473.2493 | 473.2449 | HN 1-4 | Fiber Only |
| 9.14 | 472.2758 | 472.2760 | BID 92-95 | Yes |
| 9.38 | 445.2649 | 445.2659 | BID 195-198 | Yes |
| 10.57 | 435.1424 | 436.2442 | BID 199-202 | Yes |
| 12.31 | 4972.6114 | 1243.5604 (M4H+) | BID 122-164 | Yes, stronger in Fiber |
| 13.87 | 1227.5666 | 1227.5758 | BID 96-106 | Yes |
| 15.12 | 1503.7053 | 1503.7120 | BID 79-91 | Yes |
| 15.46 | 1079.6087 | 1079.6074 | BID 166-175 | Yes, stronger in Control |
|  | 1207.7037 | 1207.7020 | BID 165-175 | Yes, stronger in Control |
| 17.80 | 1957.9705 | 1957.9651 | BID 79-95 | Yes |
| 21.11 | 1546.9195 | 1546.9245 | BID 107-121 | Yes |
| 21.63 | 3164.5051 | 1055.5042 (M3H+) | BID 43-70 | Yes, stronger in Control |
| 23.51 | 862.3967 | 862.7651 | BID 195-202 | Yes |
| 24.35 | 2685.4822 | 2685.4744 | HN 1-24 | Fiber Only |
| 24.75 | 4848.1663 | 1617.3898 (M3H+) | BID 1-42 | Yes, stronger in Control |
| 24.86 | 2003.1125 | 2003.1136 | HN 5-22 | Fiber Only |
| 25.66 | 1847.0114 | 1847.0076 | HN 5-21 | Fiber Only |
|  | 2302.2429 | 2302.2358 | HN 1-21 | Fiber Only |
| 25.76 | 1700.9097 | 1700.9099 | BID 133-147 | Yes, stronger in Control |
| 25.98 | 1184.7567 | 1184.7584 | BID 154-164 | Yes, stronger in Bid Only |
| 26.38 | 8554.2993 | 8554.3755 | BID 1-75 | Fiber Only |
| 26.82 | 7999.7361 | 7999.9959 | BID 1-70 | Yes |
| 29.47 | 4936.8644 | 1645.9015 (M3H+) | BID 148-190 | Yes |
| 30.31 | 5456.4654 | 5456.5033 | BID 148-194 | Yes, stronger in Control |
|  | 7966.1404 | 7966.1317 | BID 122-190 | Yes, stronger in Fiber |
| 30.76 | 7507.6658 | 7507.6420 | BID 126-190 | Yes, stronger in Fiber |
| 31.36 | 6301.4123 | 6301.2691 | BID 148-202 | Yes, stronger in Control |
|  | 8027.2669 | 8027.2188 | BID 126-194 | Yes, stronger in Fiber |
| 31.9 | 14011.9552 | 14011.9719 | BID 79-202 | Yes, stronger in Control |
| 32.49 | 8454.7708 | 8454.8811 | BID 126-198 | Fiber Only |

**Table S2.** Summary of deconvoluted mass spectra for each peak observed in the chromatograms of Figure 2A. Comparison of theoretical values and measured values.

| **[Humanin] (μM)** | **HN-C8A #1** | **HN-C8A #2** | **HN-C8A #3** |
| --- | --- | --- | --- |
| 0 | 3996.921 | 3392.556 | 3419.655 |
| 2.5 | 5821.271 | 6494.799 | 5648.929 |
| 5 | 8654.086 | 7633.521 | 8479.516 |
| 10 | 12155.45 | 12377.74 | 10838.49 |
| 15 | 13910.85 | 15385.97 | 13789.57 |
| 20 | 15297.1 | 16575.78 | 16861.35 |
| 35 | 16873.87 | 20754.22 | 17002.43 |
| 50 | 19710.24 | 20836.73 | 19657.34 |

**Table S3A** Raw photon count datapoints for the HN-C8A curve in the light scattering graph of Figure 3A.

| **[Humanin] (μM)** | **HN-S14G #1** | **HN-S14G #2** | **HN-S14G #3** |
| --- | --- | --- | --- |
| 0 | 3136.679 | 4351.649 | 2856.363 |
| 2.5 | 12883.01 | 12674.02 | 12335.25 |
| 5 | 21358.35 | 21895.38 | 18468.82 |
| 10 | 24894.45 | 27081.74 | 20197.17 |
| 15 | 25859.85 | 26480.08 | 21121.06 |
| 20 | 26580.97 | 25729.74 | 22018.32 |
| 35 | 31191.48 | 29996.82 | 25732.45 |
| 50 | 34615.23 | 33733.66 | 32422.18 |

**Table S3B** Raw photon count datapoints for the HN-S14G curve in the light scattering graph of Figure 3A.

| **[Humanin] (μM)** | **vMIA #1** | **vMIA #2** | **vMIA #3** |
| --- | --- | --- | --- |
| 0 | 4286 | 3792 | 3511 |
| 2.5 | 4979 | 5940 | 3835 |
| 5 | 5319 | 3996 | 4697 |
| 10 | 5623 | 4707 | 4633 |
| 15 | 5413 | 4599 | 4930 |
| 20 | 5777 | 4480 | 4351 |
| 35 | 5402 | 4760 | 4504 |
| 50 | 5669 | 4616 | 4497 |

**Table S3C** Raw photon count datapoints for the vMIA curve in the light scattering graph of Figure 3A.

|  | **ThT Fluorescence Response** | | |
| --- | --- | --- | --- |
| **Sample Number** | **1** | **2** | **3** |
| **Buffer Only** | 2427.46 | 2380.76 | 2488.83 |
| **5 µM BID** | 2307.39 | 2295.37 | 2443.47 |
| **50 µM WT HN** | 4099.67 | 3719.25 | 3878.07 |
| **50 µM HN-C8A** | 2710.34 | 2723.69 | 2993.23 |
| **50 µM HN-S14G** | 5217.14 | 5063.58 | 4931.39 |
| **50 µM vMIA** | 2232.67 | 2380.76 | 2311.38 |
| **BID & WT HN** | 35499.12 | 34811.94 | 34661.93 |
| **BID & HN-C8A** | 3939.49 | 3707.22 | 3728.59 |
| **BID & HN-S14G** | 11342.28 | 11960.05 | 12893.90 |
| **BID & vMIA** | 2207.32 | 2247.35 | 2222.00 |

**Table S4** Raw photon count datapoints for the ThT response graph in Figure 3B. The average of each datapoint was subtracted from the average of the buffer control and then the mixture response was subtracted by the peptide-only response to report the change in fluorescence upon addition of BID.
